# Droplet Squeeze Microfluidic Platform for Generating Extracellular Vesicle Hybrids for Drug Delivery

**DOI:** 10.1002/smll.202503807

**Published:** 2025-08-07

**Authors:** Uday Chintapula, Shujing Liu, Andres Fernandez Del Castillo, Jianhua Lim, Yoonho Roh, Shrawan Kumar Mageswaran, Xiaogang Zhang, Renee‐Tyler T. Morales, Mark A. Sellmyer, Yi‐Wei Chang, Xiaowei Xu, Jina Ko

**Affiliations:** ^1^ Department of Pathology and Laboratory Medicine University of Pennsylvania Philadelphia PA 19104 USA; ^2^ The Department of Biochemistry and Biophysics Perelman School of Medicine University of Pennsylvania Philadelphia PA 19104 USA; ^3^ Department of Bioengineering University of Pennsylvania Philadelphia PA 19104 USA; ^4^ Department of Energy and Chemical Engineering Incheon National University 119 Academy‐ro, Yeonsu‐gu Incheon 22012 Republic of Korea; ^5^ Department of Radiology Perelman School of Medicine University of Pennsylvania Philadelphia PA USA

**Keywords:** cancer, droplet microfluidics, drug delivery, EV hybrids, extracellular vesicles, lipid nanoparticles, melanoma

## Abstract

Extracellular vesicles (EVs) are emerging as versatile drug delivery systems due to their intrinsic biocompatibility and targeting capabilities. However, EV integrity and efficient drug loading challenges hinder their clinical translation. To address these limitations, hybrid systems integrating lipid nanoparticles (LNPs) with EVs have gained attention for their potential in targeted and combinatorial drug delivery. This study presents a robust microfluidic approach for the scalable generation of drug‐loaded EV‐LNP hybrids (EV hybrids). The method facilitates controlled fusion between EVs and LNPs by utilizing a droplet‐mediated squeezing mechanism. Lipid composition and microfluidic parameters are optimized for the fusion of EVs and LNPs and determined physicochemical and functional characterizations of the EV hybrids. In vitro studies demonstrate that EV hybrids exhibit enhanced targeting efficiency. Moreover, small‐molecule therapeutics are successfully encapsulated within EV hybrids, significantly improving cytotoxic efficacy against melanoma in 2D and 3D culture models compared to drug‐loaded EVs or LNPs alone. The work introduces a scalable, minimally disruptive microfluidic platform for engineering EV hybrids, offering a promising strategy to advance precision nanomedicine.

## Graphical abstract

A microfluidic droplet squeezing platform enables efficient fusion of extracellular vesicles with lipid nanoparticles (LNPs), generating hybrid nanocarriers with enhanced cargo loading and delivery potential. The shear‐induced mechanism within confined droplets improves fusion efficiency, providing a scalable and tunable strategy for engineering extracellular vesicle hybrids for targeted drug and gene delivery applications.

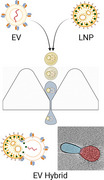

## 1 Introduction

LNPs have shown immeasurable success as drug delivery vehicles.^[^ ^1^, ^2^ ^]^ Small molecules and nucleic acids (e.g., small interfering RNA (siRNA), messenger RNA (mRNA), and plasmids) are susceptible to enzymatic degradation and have low bioavailability and targeting, leading to various side effects. LNPs provide several benefits to improve the bioavailability of these drugs by encapsulating them in materials such as lipids, controlling drug release, and offering engineering for targeting abilities.^[^ ^3^, ^4^ ^]^ Despite their promising potential, LNPs still face several limitations in drug delivery applications to specific targets.^[^ ^3^, ^5^ ^]^ Even with sophisticated LNP systems, such as the use of ionizable lipids and targeting moieties, they still face toxicity issues, adverse clinical effects, and low therapeutic outcomes due to their clearance.^[^ ^6^ ^]^ Furthermore, achieving precise targeting and controlled release of the therapeutic agent remains challenging, necessitating ongoing research to optimize these delivery systems.

Extracellular vesicles (EVs) are naturally occurring, membrane‐bound particles released by cells that have emerged as promising vehicles for drug delivery.^[^ ^7^ ^]^ Due to their biocompatibility, ability to evade the immune system, and inherent capacity to deliver therapeutic cargo such as proteins, RNA, and small molecules directly to target cells, EVs offer significant advantages over synthetic material‐based drug delivery systems and cell membrane‐coated nanoparticles.^[^ ^7^, ^8^, ^9^, ^10^, ^11^ ^]^ Similarly, cell membrane‐coated nanoparticles have been explored for targeted drug delivery due to their potential for immune evasion and tissue‐specific targeting. However, their application is constrained by inconsistencies in membrane coating techniques and the absence of intrinsic bioactive components characteristic of EVs.^[^ ^12^ ^]^ While EVs face challenges such as heterogeneous populations and limited drug loading capacity, their integration with lipid nanoparticles (LNPs) offers a synergistic platform that overcomes these limitations and provides superior therapeutic potential compared to cell membrane coating alone. Various methods are explored to improve drug loading, with potential disruption of EV integrity during the loading processes.^[^ ^13^ ^]^ Various methods have been explored to load cargo into EVs, with very few reaching clinical trials, which exhibits the low translational efficacy of EV‐loaded cargo.^[^ ^14^, ^15^ ^]^ New methods of loading small‐molecule drugs without interfering with EV integrity are needed to exploit the therapeutic effects of EV targeting and immunomodulation.

EV hybrids consisting of LNPs and EVs offer several advantages. These include enhanced targeting specificity and reduced off‐target effects due to their natural ability to home in on specific tissues or cells.^[^ ^16^, ^17^, ^18^ ^]^ These hybrids combine the biocompatibility and low immunogenicity of natural EVs with the customizable functionality (e.g., cargo, surface membrane) of synthetic LNPs. Therefore, a robust and scalable approach to making EV hybrids can improve their utility and clinical translation. Currently, various physical and chemical methods are employed to synthesize EV hybrids. Physical methods include freeze‐thawing, sonication, and extrusion of liposomes with EVs of interest.^[^ ^16^ ^]^ Chemical methods involve changes in lipid composition, transfection agents, pH gradients, and complementary affinity molecules.^[^ ^16^, ^19^ ^]^ These methods are limited to small molecules with low drug loading and potentially interfere with the integrity of the EV membrane and leakage of bioactive components present in the lumen.^[^ ^18^, ^20^, ^21^ ^]^ Overall, various strategies to synthesize EV hybrids have been reported, involving diverse compositions and complex and time‐consuming methods. They have poor characterization and standardized protocols, eventually curbing their clinical translation.

Few microfluidic technologies have been applied to generate hybrid vesicles combining EVs with LNPs or other synthetic materials.^[^ ^22^, ^23^, ^24^ ^]^ Techniques such as herringbone micromixers, acoustic microfluidics, and sonication‐assisted fusion have shown promise in enhancing fusion efficiency and therapeutic delivery. Similarly, microfluidic technologies are also applied to combine cell membrane material with liposomes to improve therapeutic outcomes.^[^ ^24^ ^]^ However, these methods face limitations such as disruption of EV membranes arising from sonication and the loss of potent EV cargo (nucleic acids, proteins, and other bioactive molecules), which reduces the therapeutic effect.^[^ ^21^ ^]^ Hence, a more robust microfluidic approach to reduce EV membrane disruption and enhance fusion with LNPs can improve drug delivery outcomes.

**Figure 1:**
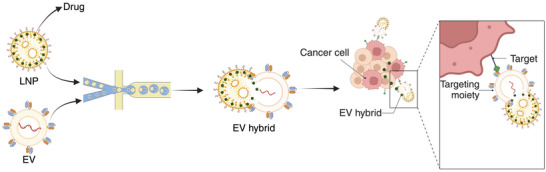
Schematic illustrating the generation of EV hybrids for targeted drug delivery.

To enable the highly efficient generation of EV hybrids with the least amount of EV modifications, we developed a microfluidic platform that enables Droplet‐Assisted Squeezing for EV Hybrids (DASH). Compared to bulk fusion or continuous‐flow methods, DASH improves vesicle uniformity and scalability. In this study, we developed a droplet microfluidic‐based method to enable a robust and straightforward approach to generating EV hybrids with high efficiency and precise control. This was achieved by utilizing charged lipids and polyethylene glycol (PEG) in LNPs and an on‐chip droplet squeezing to provide high shear rates, facilitating fusion between EVs and LNPs to form EV hybrids. Our approach enables robust fusion of EVs with LNPs carrying various cargo, without manipulating EVs, maintaining their intrinsic features. To show the feasibility of our platform for drug delivery applications, we formulated EV hybrids to target death receptor 5 (DR5) overexpressed on melanoma cells and deliver small‐molecule therapeutics (**Figure** **1**). Here we employed DR5 scFv expressing EVs isolated from NK92 natural killer cells. These cells have been well characterized and used in clinical trials, establishing their safety profile.^[^ ^25^, ^26^ ^]^ In a previous study, we also showed excellent safety profiles of NK92 EVs in vivo.^[^ ^27^ ^]^ In this proof‐of‐concept study, we showed that the EV hybrids generated by our platform enhanced cancer killing in 2D and 3D melanoma cell cultures.

## 2 Results

### 2.1 Generation and Characterization of LNPs

**Figure 2:**
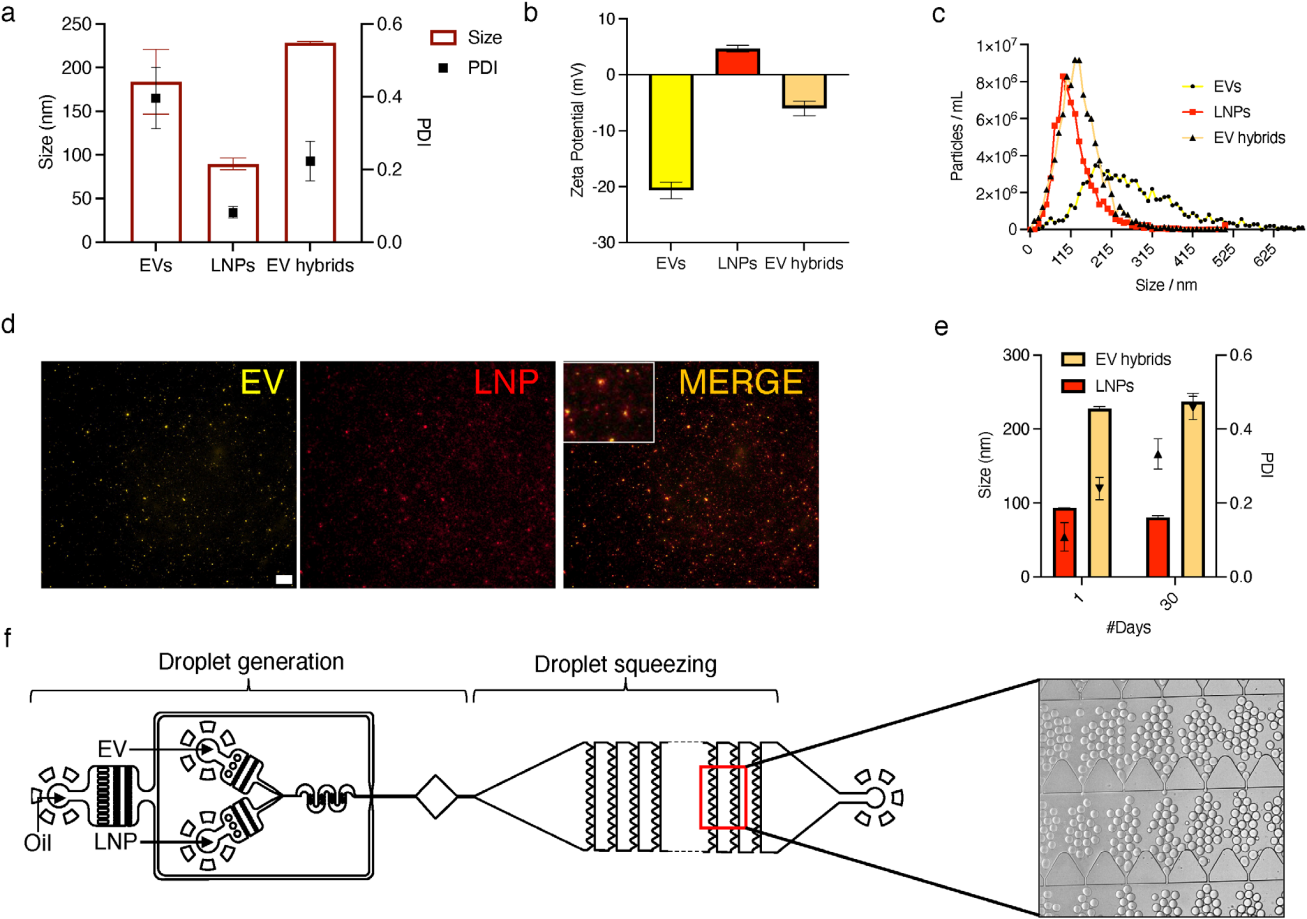
Synthesis and characterization of EV hybrids. a) DLS measurement of the size of EVs, LNPs, and EV hybrids (red bars) and PDI labeled as black squares. b) Zeta potential measurements of EVs, LNPs, and EV hybrids indicating their surface charge. c) NTA data showing the size distribution of EVs, LNPs, and EV hybrids. d) EV hybrids were imaged using a fluorescence microscope with NHS‐PEG‐AF555 labeling of EVs (yellow), Cy5 labeling of LNPs (red), and colocalized orange spots showing EV hybrids. Scale bar 10 µm. e) Stability study showing DLS data of LNPs and EV hybrids at day 1 and day 30 along with PDI represented by upright (EV hybrids) and invertied (LNPs) black arrows. f) Schematic of a droplet squeezer illustrating droplet generation with inputs for oil, EVs, and LNPs, along with droplet squeezing geometries featuring smooth, hill‐shaped structures and a microscopic image of droplets being squeezed in action. (*n* = 3, mean with standard deviation (SD) was analyzed for all samples).

Cationic lipids have been used for LNP fusion with negatively charged EV membranes.^[^ ^17^, ^28^, ^29^ ^]^ We employed DOTAP cationic lipid and neutral lipids of DPPC and cholesterol in our LNPs to facilitate fusion between LNPs and EVs (Figure S1a, Supporting Information). Additionally, the PEG molecule, which has been shown to enhance cell‐cell membrane fusion, was included in the lipid composition.^[^ ^17^ ^]^ Unlike most of the previous fusion studies using PEG in the buffer for mixing EVs and LNPs, PEG was directly embedded in the LNP membrane via DSPE‐PEG‐2000.^[^ ^17^ ^]^ We screened DSPE‐PEG‐2000 concentrations (0–10% molar ratio) in our lipid composition for LNPs and found that an increase in PEG concentration helps reduce the size of LNPs. Here, we employed a 10% molar ratio of PEG concentration, which exhibited an optimal LNP size (≈100 nm) and zeta potential (positive charge) for our fusion purposes (Figure S1b,c, Supporting Information). A microfluidic chaotic mixer using staggered herringbone geometry facilitated the synthesis of positively charged LNPs with a size of 90 ± 7 nm, a polydispersity index (PDI) of 0.09 (**Figure** **2**a), and zeta potential of 5 ± 1 mV (Figure 2b), measured by Dynamic Light Scattering (DLS). Nanoparticle Tracking Analysis (NTA) showed a uniform distribution of LNP sizes, ranging from 95 ± 20 nm with a narrow peak, in agreement with the DLS data (Figure 2c).

### 2.2 Droplet Squeezing to Generate EV Hybrids

Small EVs expressing DR5 agonist single‐chain variable fragment (ScFv) (DR5 sEVs) were engineered from CAR‐NK92 cells, as previously described.^[^ ^27^ ^]^ NK‐92 cells, which naturally produce sEVs containing cytotoxic proteins with anti‐tumor activity, were genetically modified to secrete sEVs displaying DR5‐CAR agonistic scFvs on their surface. Our western blot analysis demonstrates strong expression of EV markers CD9, CD81, and TSG101 in CAR‐NK92‐derived sEVs (Figure S2, Supporting Information). CD9, low in whole cell lysates of NK‐92 cells, was enriched in EVs, suggesting selective sorting during vesicle formation. CD81 was robustly expressed in both cells and EVs, consistent with its known role as a broadly conserved tetraspanin marker. TSG101, part of the ESCRT machinery, was detectable in EVs, confirming vesicle identity and isolation quality. Additionally, the calnexin marker was absent in EVs compared to the cell lysate, indicating that EVs were free from cell debris contamination. These findings align with prior reports of immune cell‐derived EV profiles and follow MISEV2018 guidelines.^[^ ^30^, ^31^, ^32^ ^]^ These DR5 sEVs (referred to as EVs unless specified) were subsequently fused with LNPs using a microfluidic device featuring a droplet‐squeezing geometry (Figure 2f).

The microfluidic device setup with EV and LNP inlets and collection outlet are shown in Figure S3a (Supporting Information). Smooth, hill‐shaped structures were arranged in 10 per row across 40 rows, designed to facilitate droplet squeezing at high throughput between the structures with 4 µm squeezing gaps (Figure S3b, Supporting Information). Droplets sized 40 ± 5 µm were generated using a flow‐focusing method with equal flow rates of EVs and LNPs with premixing geometry before droplet formation. A flow rate ratio of 1:5 (EV/LNP: oil) was selected for the droplet generation process. Droplet squeezing was enabled when the droplets (40 µm) traveled through the squeezing gap (4 µm). Droplets generated contained ≈34 pL of EVs and LNPs at an optimal ratio of 1:1 with an input concentration of 1–2 × 10^10^ LNP or EV/mL. High concentrations of EVs or LNPs were employed to enhance their interaction during droplet squeezing. The droplet‐generating microfluidic device design was integrated with a droplet‐squeezing region, simplifying the process into a single device. The droplets were packed with EVs and LNPs, as seen in Figure 2f and Video S1 (Supporting Information). After squeezing, the droplets were disrupted, and EV hybrids were collected by phase separation. As expected, the size of EV hybrids increased over 100 nm compared to LNP and over 45 nm compared to EV populations (Figure 2a). Zeta potential of EV hybrids was less negative (−6 mV) compared to negatively charged EVs (−20 mV), indicating the mixing of EV and LNP surface membranes (Figure 2b). Fusion was also validated from fluorescence microscopy images by showing co‐localization of AF555‐labeled EVs (yellow) and Cy5‐labeled LNPs (red), indicating EV hybrids (orange) (Figure 2d). DLS data reveal that EV hybrids and LNPs had similar average sizes over a month when stored at 4 °C, indicating the stability of the EV hybrids generated through our DASH platform (Figure 2e). However, the PDI of both LNPs and EV hybrids doubled, indicating the presence of other subpopulations of particles.

We further assessed the stability of EV hybrids via freeze‐drying. EV hybrids mixed with 8.5% w/v sucrose were lyophilized overnight and measured for size and surface charge.^[^ ^33^ ^]^ EV hybrids stored at 4 °C showed similar size and PDI compared to hybrids lyophilized and resuspended using 8.5% w/v sucrose (Figure S4a, Supporting Information). In contrast, hybrids lyophilized without sucrose as a cryoprotectant had a 3‐fold increase in size and higher PDI, indicating aggregation. Also, the zeta potential of EV hybrids with sucrose showed a similar surface charge of EV hybrids compared to those stored at 4 °C (Figure S4b, Supporting Information). Overall, EV hybrids produced in this method can be lyophilized using cryoprotectants for downstream clinical applications.

### 2.3 COMSOL Simulation of DASH Device Flow Dynamics

**Figure 3:**
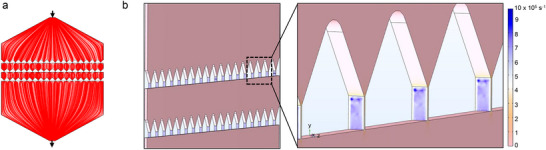
COMSOL simulation of flow in the microfluidic device. a) The model device design shows velocity profiles in red lines; boundary conditions with input velocity are equivalent to 3.3 mL h^−1^. Matching experimental condition, and pressure is zero at both input and output. b) Shear rate across the squeeze gaps, with a zoomed‐in figure illustrating the increased shear rate at the walls of the squeeze gaps. COMSOL Multiphysics software was used to generate the plots.

The shear rate has been shown to induce fusion between lipid bilayers.^[^ ^34^, ^35^ ^]^ Mechanical force‐induced stress can increase the membrane curvature stress, promoting faster and more efficient mixing and lipid membrane fusion.^[^ ^34^ ^]^ Previous studies have shown that droplet‐encapsulated cells encounter mechanical force on their membranes, making them permeable when squeezed through narrow constrictions.^[^ ^36^ ^]^ Here, we simulated the flow conditions in our droplet squeezing geometry with COMSOL Multiphysics software to assess the shear rate generated by droplet squeezing. Velocity profiles showed a reduced velocity in the microfluidic chamber compared to the inlet due to flow distribution in a widely opened chamber for squeeze parallelization (**Figure** **3**a). The shear rate was very high at the squeeze gaps, as expected in constricted spaces. Most critically, the shear rate increased to 6.5 × 10^5^ s^−1^ at the squeeze gaps compared to 0.5 × 10^5^ s^−1^ outside of the gaps (Figure 3b). Compared to conventional extrusion shear rate ranging from 100 s^−1^ to a few 1000 s^−1^, shear rate in microfluidic devices especially at unique geometries like the squeeze gaps employed in this work, is very high.^[^ ^34^, ^37^ ^]^ This high shear rate facilitates mechanical perturbations in the EVs and LNPs, including membrane permeability, membrane pore formation, and faster and more efficient mixing that encourage particle fusion. Our simulation shows a higher shear rate of our system compared to other techniques employed for membrane fusion, supporting the hypothesis of enhanced particle fusion phenomenon via droplet squeezing (**Table** **1**).^[^ ^34^, ^38^, ^39^ ^]^ To note, droplets generated with EVs and LNPs were intact under the high shear rates generated from flow rates used in the experiments.

**Table 1:** Shear rates of techniques used for lipid membrane fusion.

Device type	Shear rate generated	Effects of shear
Droplet squeezing (This work)	>6.2 × 10^5^ s^−1^	LNP/EV fusion, drug loading
Couette flow cell^[^ ^34^ ^]^	>3000 s^−1^	Membrane fusion and deformation
Rotational Viscometer^[^ ^38^ ^]^	2700 s^−1^	Increase membrane permeability
Extrusion^[^ ^39^ ^]^	100–1000′s s^−1^	Membrane pores, Liposome EV fusion
Ultra Turrax^[^ ^39^ ^]^	400 s^−1^	Increase the elastic constant

### 2.4 Optimization of Fusion using Droplet Squeezing

**Figure 4:**
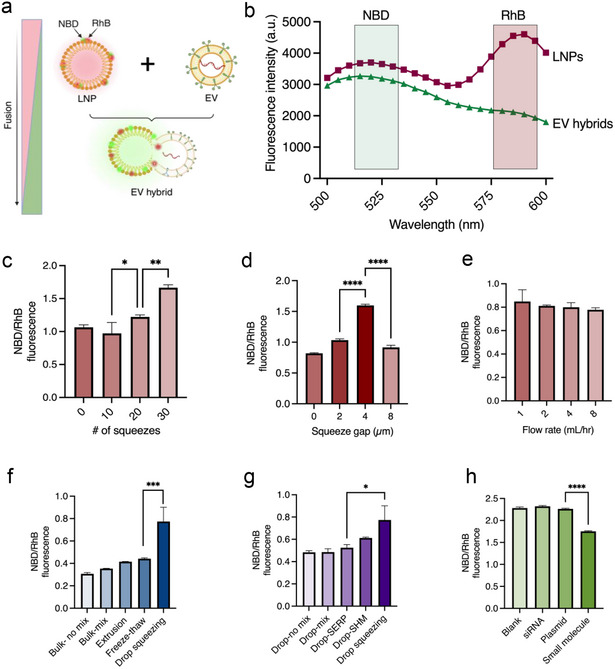
Optimization and characterization of droplet squeezing device for LNP‐EV fusion. a) The schematic illustrates the FRET dynamics wherein NBD functions as the donor and RhB serves as the acceptor molecule. LNPs incorporating this FRET pair demonstrate enhanced RhB emission (red). Upon fusion, the spatial separation of the donor and acceptor molecules leads to a significant reduction in RhB fluorescence, primarily due to the increased distance between the NBD donor, which is essential for facilitating the emission from RhB. b) Spectrophotometer reading showing NBD and RhB emission of LNPs and EV hybrids upon excitation at 460 nm (NBD excitation wavelength). Assessment of fusion activity (NBD/RhB ratio) across c) different numbers of squeezing rows, d) squeeze gaps, and e) flow rates. f) Fusion activity of droplet squeezing was compared with bulk incubation only, bulk mixing (37 °C for 1h) extrusion, and freeze‐thawing. g) Assessment of fusion activity between droplets containing EVs and LNPs within diverse microfluidic mixing architectures, including control conditions (droplet incubation only, droplet mixing), serpentine channel mixers (SERP), staggered herringbone mixers (SHM), and droplet squeezing. h) Evaluation of fusion activity upon loading of various cargos, including random siRNAs (13 kDa), EGFP plasmids (5 kb length or 3300 kDa), and small molecules (433 Da). A One‐Way ANOVA followed by Tukey's post‐hoc test was utilized to determine significance values. *n* = 3 samples with SD were analyzed for each group. ^*^ *p* < 0.05, ^**^ *p* < 0.01, ^***^ *p* < 0.001, ^****^ *p* < 0.0001.

To optimize microfluidic device parameters, LNPs were prepared with a fluorescence resonance energy transfer (FRET) pair of 2,1,3‐benzoxadiazole‐4‐yl (NBD) egg Liss Phosphatidylethanolamine (PE) as donor and egg Liss Rhodamine B (RhB) PE as acceptor molecules (Figure S1a, Supporting Information). An increase in the NBD/RhB ratio indicates reduced energy transfer between the FRET pair due to their increased distance from the fusion of the LNP membrane with EVs (**Figure** **4**a). Compared to LNP, EV hybrids showed a reduction in RhB emission and overall NBD/RhB ratio increase when excited for NBD fluorescence, confirming successful fusion (Figure 4b). The fusion activity (NBD/RhB ratio) increased with the number of squeezes and plateaued after 30 squeezes (Figure 4c). Additionally, the gap length between squeeze units influenced fusion activity, with a 4 µm squeeze gap width showing optimal fusion (Figure 4d). Interestingly, a lower 2 µm squeeze gap width did not improve the fusion activity. Different flow rates ranging from 1–8 mL h^−1^ had similar effects on the fusion process (Figure 4e).

Using the optimal microfluidic parameters, we compared droplet squeezing to various conventional fusion methods and other microfluidic geometries. Among different fusion methods, our DASH technique showed significant improvement in fusion activity compared to other conventional methods owing to the higher shear rates generated via droplet squeezing (Figure 4f). Microfluidic chaotic mixing of droplets via various geometries such as serpentine and staggered herringbone, increased fusion activity compared to plain droplet mixing, indicating the effect of a high shear rate in droplet mixing (Figure 4g). Small and large nucleic acid loading into LNPs had a minimal impact on their fusion activity with EVs. But there was a reduction of fusion activity with LNPs loaded with hydrophobic small molecule cargo, possibly due to the interference of small molecules loaded within the hydrophobic lipid membrane (Figure 4h). The EV:LNP ratio significantly influenced the synthesis of EV hybrids, with an increased proportion of LNPs enhancing the percentage of EV hybrids at EV:LNP ratios of 1:1 and 1:10. While small particle flow cytometry showed %EV hybrids plateaued beyond a 1:1 ratio, the highest degree of EV colocalization with LNPs was observed at a 1:10 ratio (Figure S5a,b, Supporting Information). Finally, we compared the fusion activity with different EV types to understand the effect of EV surface on fusion using various methods. The DASH method showed higher fusion over other methods for both suspension NK cell‐derived sEVs and adherent A431 cell‐derived sEVs (Figure S6, Supporting Information). The fusion activity in suspension NK cell‐derived sEVs was comparatively higher than adherent A431 cell‐derived EVs. Although both EVs have negatively charged surfaces, the lipid composition of suspension cell‐derived EVs contains higher amounts of PS and PE lipids, which can contribute to membrane flexibility, favoring fusion.^[^ ^40^, ^41^ ^]^ Meanwhile, adherent cell‐derived EVs have higher cholesterol and sphingomyelin content, rendering their membranes more rigid.^[^ ^40^ ^]^

### 2.5 Cryo‐Electron Tomography of EV Hybrids

**Figure 5:**
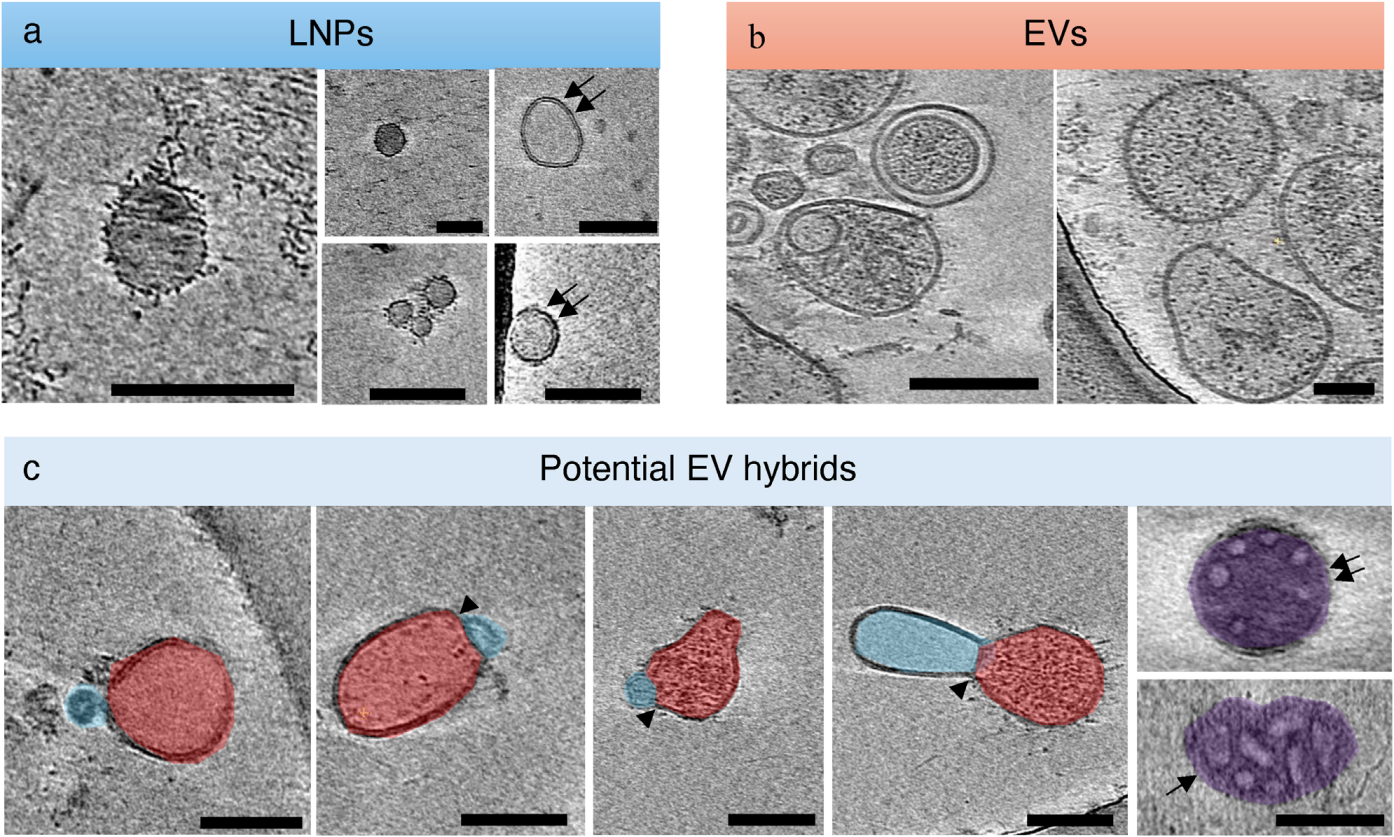
Cryo‐ET of EV hybrids. a) Representative cryo‐ET images of LNPs reveal their predominantly monolayered (and occasional bilayered) and spherical morphology. Many LNPs exhibit electron‐dense cores, indicative of successful encapsulation of the hydrophobic drug. b) EVs display heterogeneous morphologies (shapes ranging from spherical to ovoid and sizes ranging from 100 to 300 nm) with structural diversity including unilamellar and multilamellar vesicles. EVs are characterized by electron‐lucent aqueous cores and variably dense luminal inclusions. c) EV hybrids exhibit hallmark features of membrane fusion, including LNPs (blue) adhered to or fused with EV (red) membranes. In some instances (last two images in purple), the complex morphologies observed reflect potentially complex fusion mechanisms and reorganizations. Scale bar = 100 nm.

To further assess the EV and LNP fusion, we performed cryo‐electron tomography (cryo‐ET) on drug‐loaded LNPs and NK92 cell‐derived sEVs individually and after mixing them to generate EV hybrids. As a part of our analysis, we extensively surveyed our samples using high magnification (33 000X) projection images to capture the distribution of various morphologies and subsequently analyzed a representative subset by cryo‐ET. LNPs predominantly displayed classical morphology characterized by uniform, electron‐dense spherical structures with sizes ranging from 25 to 100 nm (**Figure** **5**a). They predominantly exhibited a bounding monolayer with a relatively homogeneous darker central contrast that likely indicates drug encapsulation. It is however worth noting that we occasionally observed a complete or incomplete double‐layered bounding membrane around these entities (arrow pairs). In contrast, EVs were predominantly bounded by a distinct membrane bilayer and varied in shape (from spherical to slightly ovoid) and size (mostly 100–300 nm) (Figure 5b). Their interiors contained a heterogeneous mixture of electron‐lucent regions, electron‐dense granular inclusions, and internal vesicles, consistent with known features of native EVs.^[^ ^42^ ^]^

Cryo‐ET of EV hybrids revealed several features suggestive of fusion between EVs and LNPs (Figure 5c). Across multiple tomograms, vesicle populations exhibited fusion interfaces and bilayer continuity between adjacent vesicles (arrowheads). Intriguingly, we found numerous instances of a unique encapsulated particle that possessed a dense core, interspersed with smaller electron‐lucent regions (last two panels of purple overlays). They often exhibited a double‐layered bounding membrane (arrow pair) but at other times, possessed only a single discernible bounding layer (single arrow). Importantly, these particles were not found in either of the individual samples (LNP only or EV only). Together, our observations suggest that we have captured several potential examples of EV hybrids; their varied morphology likely indicates different kinds or states of fusion. Our results are consistent with fusion‐driven integration between synthetic and biologically derived vesicles, likely facilitated by microfluidic mixing or membrane destabilization during droplet squeezing. It is noteworthy that similar cryo‐EM observations of EV and LNP fusion have been reported in previous studies.^[^ ^43^, ^44^ ^]^

### 2.6 DR5 EV Hybrid Characterization

**Figure 6:**
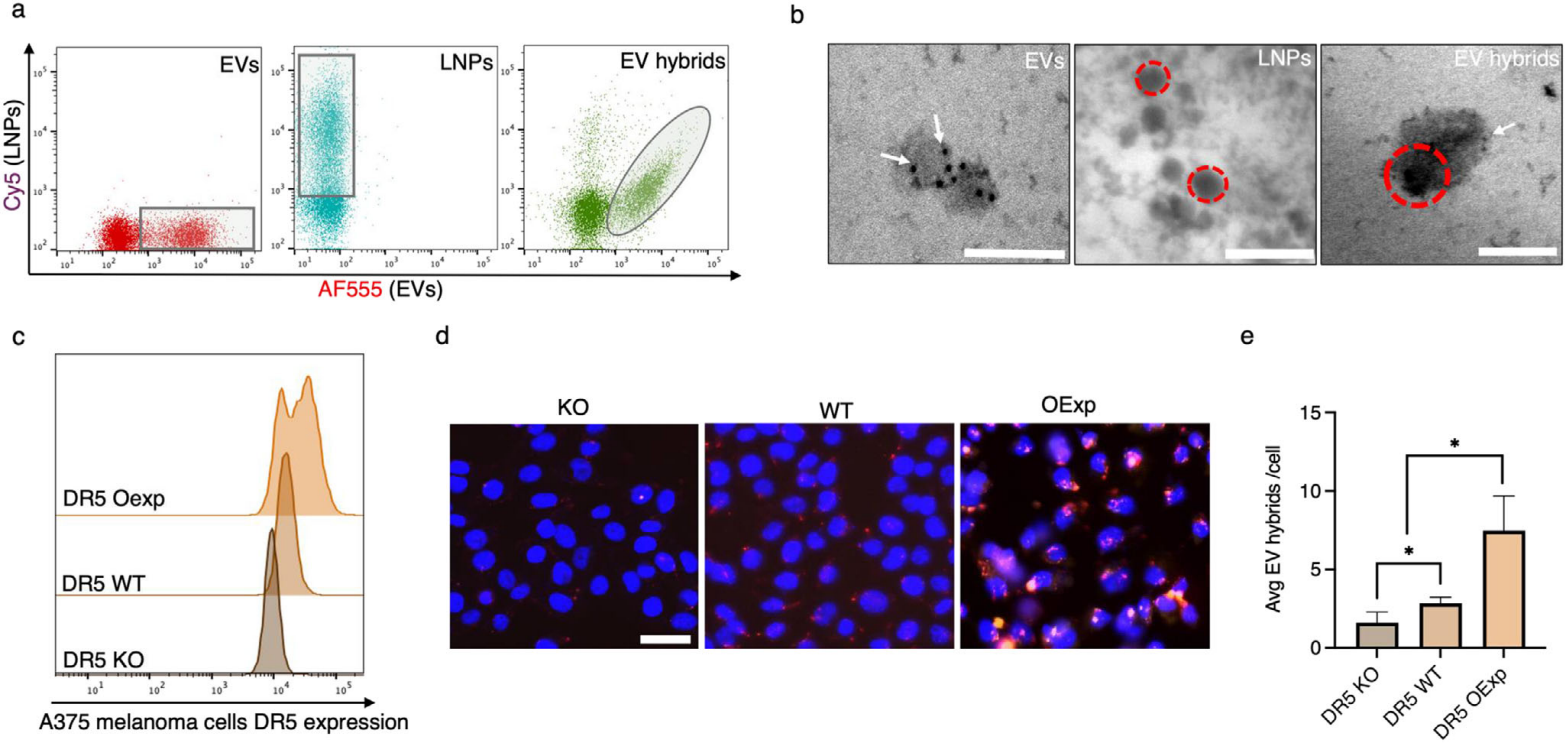
EV hybrid characterization. a) The flow cytometry analysis of small particles displays dot plots illustrating the positional shifts of AF555‐labeled EVs, Cy5‐labeled LNPs, and the double‐positive population indicative of EV hybrids. b) TEM analysis revealed AuNP‐conjugated Ab staining of DR5 scFv on EVs and EV hybrids. In contrast, LNPs appeared as distinct dark spherical entities, facilitating differentiation from the lighter‐stained EV structures. Scale bar = 200 nm. c) DR5 staining of A375 melanoma cells with various degrees of DR5 receptor expression. A clear shift is seen from low expression (KO) to increased expression of DR5 in WT and Oexp cells. d) fluorescent images showing varied cell uptake of EV hybrids in DR5 KO to higher DR5 expressing WT, and OExp cells. Nuclei are stained with DAPI for visualization. Scale bar = 20 µm. e) Quantitative analysis of EV hybrid uptake per cell in diverse melanoma cell lines expressing DR5. Paired t‐test with ^*^ *p* < 0.05 and *n* = 3 with SD.

To characterize DR5 EV hybrids, we evaluated the EV hybrids using small particle flow cytometry and electron microscopy. EV membrane proteins were labeled with NHS‐PEG4‐AF555 and fused with Cy5‐labeled LNPs using our DASH device. Flow cytometry analysis revealed that more than 65% of the detected particles were double positive (AF555+ Cy5+), indicating successful fusion of EVs and LNPs (**Figure** **6**a). Additionally, DR5 scFv antigens on EVs and EV hybrids were immunostained with anti‐DR5 antibody (Ab) and subsequently labeled using secondary Ab conjugated with 10 nm gold nanoparticles (AuNPs) for electron microscopy. Transmission electron microscopy (TEM) images of the immunostained EVs demonstrated spherical structures with an average diameter of 150–200 nm. The EVs exhibited a lighter contrast with high‐contrast AuNP staining, confirming the presence of DR5 scFv molecules on their surface (Figure 6b). In comparison, LNPs appeared as dark, spherical structures with an average size of 80–100 nm. TEM analysis of the EV hybrids showed the fusion of dark LNPs with lighter‐shaded EVs displaying AuNP labeling, further corroborating the successful fusion of EVs and LNPs.

To assess DR5 scFv functionality in EV hybrids, we stained them with anti‐F_ab_ Ab targeting DR5 scFv (Figure S7a, Supporting Information). A double‐positive population (EV hybrids) was plotted in the fluorescence channel corresponding to the anti‐F_ab_ Ab staining (Figure S7b, Supporting Information). The data show significant increases in fluorescence in EVs, and EV hybrids compared to LNPs alone, indicating the presence of functional DR5 scFv (Figure S7c, Supporting Information). Although DR5 scFv presence was observed on both EVs and EV hybrids, EVs showed higher fluorescence than EV hybrids. This may be due to fusion activity reducing the accessibility of some DR5 scFv molecules to the anti‐F_ab_ Ab. Small particle flow cytometry and TEM results reveal active DR5 scFv molecules on EV hybrids generated from our DASH device. These results indicate that our platform does not affect the integrity of the EV membrane during fusion and retains functional protein molecules on the EV hybrids which are vital for targeting.

### 2.7 Target Binding Affinity of EV Hybrids

DR5 is overexpressed in various cancers, including liver cancer, melanoma, and pancreatic cancer, while its presence in normal tissues is significantly lower.^[^ ^45^, ^46^, ^47^ ^]^ DR5 agonist scFv containing EV hybrids' binding affinity to DR5 were assessed using A375 melanoma cell lines with DR5 knockout (KO), wild type (WT), and overexpression (OExp). Labeling with anti‐DR5 Ab showed various levels of DR5 expression from least (DR5 KO) to highest (DR5 OExp) (Figure 6c). Cell uptake study shows a significantly increased uptake of EVs in WT and OExp compared to KO cells (Figure S8, Supporting Information). In contrast, LNPs showed similar uptake activity in both the KO and WT cells, indicating the affinity of DR5 scFv EVs to target DR5 (Figure S8, Supporting Information). EV hybrids showed higher uptake in WT and OExp compared with KO cells, displaying the affinity of the active DR5 scFv targeting molecules on the EV hybrids (Figure 6d). Z‐stacked images were acquired and analyzed using CellProfiler 4 software to assess the colocalization and uptake of EV hybrids (Figure S9, Supporting Information).^[^ ^48^ ^]^ The custom pipeline analysis considered key factors, including EV and LNP colocalization within each cell, exclusion of large aggregates due to increased frame thickness, and removal of small pixels to minimize false positives. Quantitative data compiled from Z‐stacked images of the mid‐cell region revealed a significant uptake of EV hybrids in DR5 WT and OExp cells compared to DR5 KO cells (Figure 6e).

### 2.8 EV Hybrids as a Drug Delivery System against Melanoma

**Figure 7:**
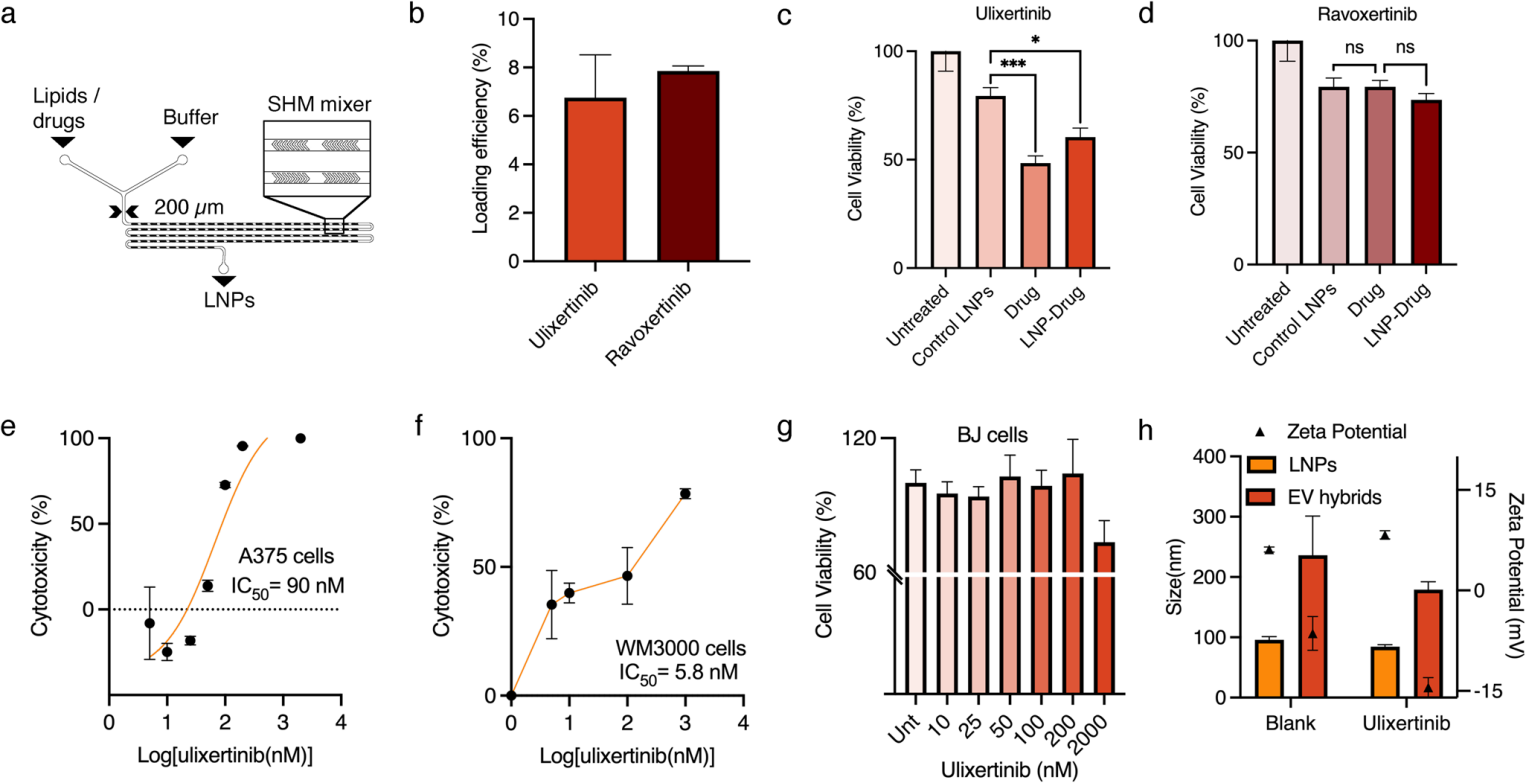
Characterization of drug‐loaded LNPs. a) Schematic of microfluidic device with SHM design used to synthesize drug‐loaded LNPs. b) LC‐MS measurement of drug loading efficiency in lysed LNPs. c) Cell viability of A375 melanoma cells after treatment with 100 nm ulixertinib as free drug or drug‐loaded LNPs (LNP‐Drug), measured from luciferase activity. d) Cell viability of A375 melanoma cells after treatment with 100 nm ravoxertinib as free drug or LNP‐Drug, measured from luciferase activity e) IC_50_ calculation of ulixertinib in A375 melanoma cells (10‐2000 nm), measured from luciferase activity. f) IC_50_ calculation of ulixertinib in WM3000 NRAS mutant metastatic melanoma cells (10–1000 nm), measured with MTS reagent for cell viability. g) Cell viability of BJ fibroblast cells upon treatment with ulixertinib (10–2000 nm), measured with MTS assay. h) DLS measurement of size, zeta potential, and PDI of blank and ulixertinib‐loaded LNPs and EV hybrids generated from each one. *n* = 3 with SD. A one‐way ANOVA followed by Tukey's post‐hoc test was utilized to determine significance values. *n* = 3 samples with SD were analyzed for each group. *p < 0.05, ***p < 0.001

Given the enhanced affinity of EV hybrids for DR5‐expressing cells, we studied their potential as drug delivery vehicles, specifically for targeting DR5‐expressing melanoma cells and delivery of cancer therapeutics. ERK inhibitors are currently used in clinical trials to treat various MAPK pathway‐driven cancers such as melanoma, colorectal, thyroid, and lung cancer.^[^ ^49^, ^50^, ^51^, ^52^ ^]^ Although these drugs are highly potent against cancer cells, they still present toxicities in clinical studies.^[^ ^53^ ^]^ Dermatologic adverse effects and other adverse events observed in the clinical trials can be explained by the involvement of ERK 1/2 activity across various physiological processes, including those in the gastrointestinal tract and skin.^[^ ^54^, ^55^, ^56^ ^]^ We employed two ERK 1/2 inhibitors, ulixertinib and ravoxertinib, as our cargo loaded into the LNPs, to enable combinational targeted delivery with EV hybrids.^[^ ^53^, ^57^ ^]^ Each hydrophobic drug was mixed with lipids in ethanol and later mixed with PBS in a microfluidic SHM design to generate homogeneous drug‐loaded LNPs (**Figure** **7**a). Loading efficiencies for ulixertinib and ravoxertinib were 6.3% and 8%, respectively, with both drug concentrations at ≈40 µg mL^−1^ in the LNPs, measured from lysed samples via Liquid Chromatography‐Mass Spectrometry (LC‐MS) (Figure 7b). Drug release kinetics of similar hydrophobic drugs from liposomes show release of over 80% of the drugs within 2 days.^[^ ^24^ ^]^ Both drugs have high partition coefficient (ulixertinib = 3.6, ravoxertinib = 2.7) toward octanol, complicating the use of perfluorooctanol (PFO) for droplet disruption and EV hybrid collection. To overcome this challenge, we employed an anti‐static gun to disrupt the droplets, effectively preventing drug loss or leakage into PFO.

To evaluate drug bioavailability following incorporation into LNPs, we assessed the cytotoxicity of drug‐loaded LNPs in WT A375 melanoma cells. At a drug concentration of 100 nM, ulixertinib‐loaded LNPs exhibited cytotoxic effects comparable to the free drug, whereas ravoxertinib‐loaded LNPs showed no significant difference from control LNPs, suggesting that at similar concentrations, ravoxertinib has poor bioavailability when delivered via LNPs (Figure 7c,d). Based on these cytotoxicity results, ulixertinib‐loaded LNPs were selected to prepare EV hybrids for subsequent drug delivery studies. Concerning drug release, previous studies indicate a sustained release of hydrophobic drugs from both LNPs and the hybrids generated from them.^[^ ^24^ ^]^ Based on the reported data, EV hybrids have a slightly faster drug release compared to LNPs.^[^ ^24^, ^58^ ^]^ Systematic exploration of lipid compositions in LNPs and EV membrane composition is needed to understand drug release mechanisms in EV hybrids. In particular, the DASH platform, by enabling controlled fusion of EVs, offers a robust framework for tuning drug release profiles in EV hybrids.

To determine the optimal dosage for drug delivery and assess biocompatibility, free ulixertinib was given to control BJ fibroblast cells, WT A375 melanoma cells, and WM3000 metastatic melanoma cells with NRAS mutation at varying concentrations. Ulixertinib demonstrated significant cytotoxicity in melanoma cells, reducing cell viability by 50% at 90 nm in WT A375 cells (Figure 7e) and at 5.8 nm in WM3000 cells (Figure 7f), while exhibiting minimal cytotoxic effects in BJ cells up to 2000 nm (Figure 7g). Based on these findings, a concentration range of 100–500 nm ulixertinib was selected for subsequent evaluations of EV hybrids in melanoma‐targeted drug delivery. To make drug‐loaded EV hybrids, ulixertinib‐loaded LNPs were fused with DR5 EVs at a 1:1 ratio using our DASH technique. DLS measurements showed a two‐fold increase in the size of LNPs and a change in charge from +8 mV to −12 mV, indicating a fusion of EVs and drug‐loaded LNPs (Figure 7h). Similarly, ravoxertinib‐loaded LNPs when fused with DR5 EVs showed a change in charge from +6.7 mV to −15 mV and a three‐fold increase in size of LNPs was observed after fusion with EVs, indicating the utility of the DASH technique to work with various drugs (Figure S10, Supporting Information).

### 2.9 Therapeutic Potential of EV Hybrids in 2D and 3D Melanoma Cell Cultures

**Figure 8:**
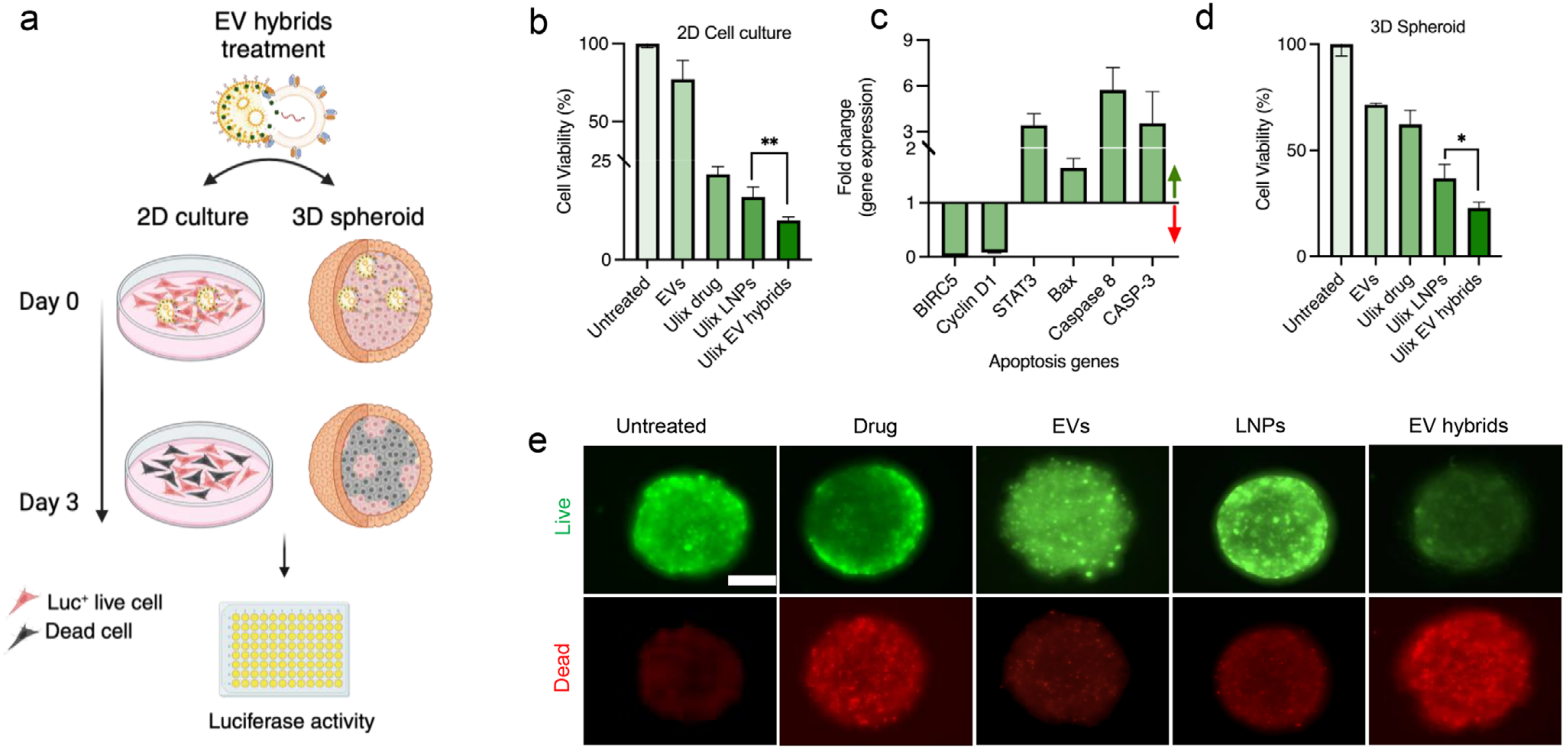
Therapeutic potential of EV hybrids as a drug delivery system. a) Schematic of in vitro treatment of EV hybrids in a 2D cell culture and 3D spheroids. b) Cell viability of luciferase^+^ A375 melanoma cells cultured in a 96‐well plate treated with ulixertinib‐loaded EV hybrids (ulix EV hybrids) for 3 days. 500 nm ulixertinib was used across all groups. c) Gene expression of apoptotic genes generated from qPCR with RNA extracted from EV hybrid‐treated A375 melanoma cells. d) Cell viability of luciferase^+^ A375 melanoma cells in 3D spheroids after treatment with 2.5 µm ulixertinib as a free drug (ulix drug) or loaded in LNPs and EV hybrids. e) LIVE/DEAD staining of 3D spheroids shows the reduction in GFP expressing A375 melanoma cells and an increase in dead cells after 3 days of treatment with EV hybrids. BJ fibroblast cells in the spheroids do not have GFP expression. Dead cells were visualized with propidium iodide staining. Scale bar = 200 µm. Ulixertinib is represented by ulix. A one‐way ANOVA followed by Tukey's post‐hoc test was utilized to determine significance values. *n* = 3 samples with SD were analyzed for each group. ^*^ *p* < 0.05, ^**^ *p* < 0.01.

ERK inhibitors show promise in melanoma therapy but face toxicity challenges in clinical trials.^[^ ^53^ ^]^ To mitigate this, we explored EV hybrids for targeted ulixertinib delivery. The treatment efficacy of EV hybrids was studied in 2D melanoma cell culture and 3D melanoma sphreoids. As a critical control to mimic EV hybrids, anti‐DR5 Ab conjugated LNPs were prepared using maleimide‐thiol chemistry (Figure S11a, Supporting Information). Staining of LNPs with anti‐human IgG secondary Ab showed successful anti‐DR5 Ab conjugation onto LNPs with an optimal molar ratio of 2.5:1 (Ab: maleimide) (Figure S11b,c, Supporting Information). To evaluate their therapeutic efficacy, ulixertinib‐loaded EV hybrids and LNPs were given to both 2D and 3D melanoma cell models with luciferase^+^ DR5 overexpressing A375 cells (**Figure** **8**a). In 2D cultures treated with an equivalent drug concentration of 500 nm across all groups, ulixertinib‐loaded EV hybrids demonstrated significantly lower cell viability (10%) compared to drug‐loaded LNPs (16%), free drug (22%), and native EVs (77%) (Figure 8b). Notably, while NK cell‐derived sEVs inherently exert an inhibitory effect on melanoma cells, the combination EV hybrids exhibited increased cytotoxicity compared to anti‐DR5 Ab conjugated ulixertinib‐loaded LNPs and ulixertinib‐loaded EVs (Figure S12a, Supporting Information). This enhanced therapeutic efficacy is likely attributable to a combinatorial approach involving direct ulixertinib‐mediated cytotoxicity, selective engagement of DR5 by the DR5 agonist scFv to trigger apoptotic signaling, and the presence of cytotoxic components within the EV lumen—an advantage absent in anti‐DR5 Ab conjugated LNPs.^[^ ^27^, ^59^ ^]^ Supporting this data, prior studies have demonstrated that NK cell‐derived sEVs harbor cytotoxic proteins and intrinsic anti‐tumor properties, potentiating melanoma cell death and modulating the tumor microenvironment.^[^ ^27^ ^]^

Further RNA analysis of EV hybrid‐treated 2D cell cultures revealed differential expression of key apoptosis‐related genes, including Caspase‐8, Caspase‐3, STAT‐3, BIRC5, Bax, and Cyclin D1 (Figure 8c). Gene expression levels were normalized to the reference gene of Ribosomal Protein L13a (RPL13A). Notably, the anti‐apoptotic genes such as Cyclin D1 and BIRC5 were downregulated following EV hybrid treatment, suggesting effective apoptosis. This observation aligns with previous studies demonstrating Cyclin D1 downregulation upon ulixertinib treatment, indicating ERK pathway inhibition as a contributing mechanism.^[^ ^60^ ^]^ Consistently, our analysis revealed a significant downregulation of BIRC5 and Cyclin D1 across all drug‐loaded groups, confirming efficient intracellular drug release from LNPs and EV hybrids (Figure S12b, Supporting Information). Additionally, pro‐apoptotic genes, including Caspase‐8, Caspase‐3, and Bax, were upregulated following EV hybrid treatment, supporting an apoptotic‐driven mechanism of action. This result is consistent with previous findings where ulixertinib treatment led to increased expression of Caspase‐3 and Bax.^[^ ^61^ ^]^ Note that the STAT3 gene was upregulated across all drug‐related groups. A comparable apoptotic gene expression profile was observed across free ulixertinib, drug‐loaded LNPs, and EV hybrid treatment, further validating the robustness of our DASH method to generate EV hybrid drug delivery systems (Figure S12b, Supporting Information).

We further studied the ability of EV hybrids to deliver cargo in a 3D spheroid model composed of GFP^+^ A375 melanoma cells and BJ fibroblast cells. EV hybrids showed significantly lower cell viability (22.8%) of melanoma cells in the spheroids compared with drug‐loaded LNPs (36.1%) or EVs alone (71.3%) (Figure 8d). Moreover, EV hybrid treatment exhibited significant cytotoxicity against DR5‐expressing melanoma cells compared to anti‐DR5 Ab‐conjugated and ulixertinib‐loaded LNPs at a drug concentration of 2.5 µm (Figure S12c, Supporting Information). Reduction in GFP^+^ A375 melanoma cells in spheroid cultures further confirmed cytotoxic effects within the central layers, suggesting effective penetration of EV hybrids into the spheroid structure (Figure 8e). Propidium Iodide staining of spheroids shows an increased number of dead cells in EV hybrid treatment compared to other groups. These findings highlight the potential of the DASH technique as a powerful platform for generating customizable EV hybrids with superior therapeutic capabilities.

EV hybrids loaded with ulixertinib exhibited enhanced cytotoxicity against A375 melanoma cells compared to LNPs or EVs alone. To delineate the source of this increased efficacy, we compared EV hybrids to a combination treatment of free ulixertinib and anti‐DR5 Ab, which engages with the TRAIL pathway to induce apoptosis. Our results demonstrate that EV hybrids outperform this combination, indicating that the therapeutic effect is not solely due to DR5‐mediated targeting or drug synergy, but also influenced by the intrinsic bioactive cargo of NK cell‐derived sEVs employed (Figure S13, Supporting Information).^[^ ^27^ ^]^

## 3 Discussion

Recent studies have explored the fusion of LNPs with EVs to enhance drug loading and profiling of EV cargo.^[^ ^62^, ^63^ ^]^ To overcome the challenges of conventional methods in preparing EV hybrids, we present the first proof‐of‐concept for utilizing droplet squeezing to facilitate LNP and EV fusion.^[^ ^28^, ^29^, ^64^ ^]^ Our device successfully generated EV hybrids using different EV sources and encapsulated various cargos within the EV hybrids, including plasmids, small RNAs, and drugs. Unlike most strategies reported for EV fusion, the DASH technique eliminates the requirement for pre‐processing of EVs with tags or affinity‐binding molecules, preserving the integrity of the EV membrane and its internal bioactive components critical for therapeutic applications.^[^ ^65^ ^]^ Maintaining the pristine nature of EVs, the DASH technique can be extended as a minimally disruptive method for profiling EV cargo. This platform can also be applied to generate various barcoded EV hybrids for EV profiling and screening of EV targets for organ‐specific delivery and blood‐brain barrier crossing.^[^ ^66^ ^]^

While our technology offers many advantages, there are some limitations that can be addressed to further enhance its performance. Despite the optimization of LNPs for a positive charge in favoring fusion, screening for other lipids to enhance endosomal escape can improve the overall delivery of cargo after cell uptake. The DASH technology enables the screening of various lipid compositions, enhancing organ‐specific delivery of EV hybrids. Although the technique achieved a fusion efficiency of >65%, further enrichment through density gradient centrifugation can improve the purity of EV hybrids for clinical applications.^[^ ^67^ ^]^ Additionally, simulations that include nanoparticles inside a droplet and their fusion behavior across squeezing geometries at various flow rates can help understand the driving factors of fusion (e.g., geometries, size, ion concentrations, etc.) in the microfluidic device. Mechanistic cell uptake and drug delivery features of EV hybrids need further studies to improve drug release kinetics. While our studies demonstrate the stability of EV hybrids in terms of average size over a month by refrigeration and freeze drying using sucrose, longitudinal lyophilization studies can further enhance their stability and clinical translational potential.^[^ ^68^ ^]^ Overall, our microfluidic DASH technology, which harnesses a droplet‐squeezing approach, holds promise for scalable, high‐throughput production of EV hybrids, paving the way for diverse diagnostic and therapeutic applications.

In future work, we aim to enhance the platform by integrating parallelized droplet generation to increase throughput, enabling the scalable production of EV hybrids for improved clinical translation.^[^ ^69^ ^]^ Additionally, we will investigate the delivery of nucleic acids using EV hybrids, with a particular focus on targeted gene therapy applications. We will systematically evaluate key droplet parameters, including viscosity, buffer pH, and concentration‐dependent effects on the fusion process to further optimize our platform. Furthermore, we plan to explore the use of diverse EV subtypes, including small and large EVs secreted by various cell types, to expand the applicability of our approach in generating tailored EV hybrids for therapeutic applications.

## 4 Experimental Section

### Cell Culture

A431 cell line was purchased from the American Type Culture Collection and CAR‐NK cell producing DR5 scFv expressing EVs were previously developed in the Xu lab.^[^ ^27^ ^]^ A431 cells were maintained in Dulbecco's modified Eagle's medium (DMEM), supplemented with 10% fetal bovine serum (FBS), 100 IU penicillin, and streptomycin (100 µg mL^−1^) (Pen‐Strep). CAR‐NK92 cells were maintained in a modified RPMI media containing 10% FBS, 10% horse serum, 0.2 mm myo‐inositol, 0.1 mm 2‐mercaptoethanol, 0.02 mm folic acid, recombinant human IL‐2 (200 U mL^−1^), and 1% penicillin‐streptomycin at 37 °C and 5% CO_2_. WM3000, and BJ cells were cultured in DMEM supplemented with 10% FBS and 1% Pen‐Strep.

### EV Isolation and Characterization

CAR‐NK92 and A431 cells were cultured in the medium as described in the previous section, but FBS and horse serum were replaced with 5% EV‐depleted serum once cells reached a confluency of 70–80%. After 48 hrs, supernatants were centrifuged at 2000 g for 15 min to remove cells and debris, followed by centrifugation at 10 000 g for 30 min to remove microvesicles. Later, the supernatant was centrifuged at 120 000 g for 2 h at 4 °C. Pelleted sEVs were resuspended in PBS and stored at −80 °C in aliquots until further experiments.

After isolating EVs, the samples were analyzed using two methods. The protein concentration was determined with the Qubit system (Thermo Fisher), following the manufacturer's protocol with the Qubit protein assay kit. Additionally, the particle count was assessed using NTA. The NTA measurements were performed using the ZetaView PMX220 Twin instrument (Particle Metrix) at the Extracellular Vesicle Core of the University of Pennsylvania School of Veterinary Medicine. The analysis was conducted with a sensitivity setting of 70.

### Western Blot

NK92 cells or CAR‐NK92 sEVs were lysed in radioimmunoprecipitation assay (RIPA) buffer with Protease inhibitor cocktail. A total of 10–20 µg of protein was processed for SDS‐PAGE and electroblotted onto polyvinylidene difluoride (PVDF) membranes (Invitrogen, USA). Blots were blocked with 5% nonfat milk and incubated with corresponding primary antibodies (anti‐CD9, CD81, Calnexin, and TSG101) and horseradish peroxidase (HRP)‐conjugated secondary antibodies (BioLegend, USA). Membranes were developed using enhanced chemiluminescence (ECL) detection reagents (Pierce, Thermo Scientific, MA, USA).

### Antibodies

Anti‐DR5 monoclonal antibodies (tigatuzumab) were manufactured by Biointron Biological USA Inc. (Metuchen, NJ, USA). The molar ratios and number of antibody molecules needed for LNP conjugation was calculated using the surface area of the LNP and Avogadro's number. For western blot, antibodies of CD9 (BioLegend, Cat #31202), CD81 (BioLegend, Cat #349502), Calnexin (BioLegend, Cat #699401), and TSG101 (BioLegend, Cat #934302) were used.

### 3D Spheroid Formation

50 µL of 1.5% agarose solution was used in coating 96‐well plates before seeding cells. After 1hr incubation, WT A375 melanoma cells (20 000/well) and BJ cells (40 000/well) were mixed and seeded. This cell mixture was incubated for 48 h for spheroids to form.

### LNP Synthesis

Lipids for LNP synthesis were purchased from Avanti polar lipid (USA). 1,2,‐dioleoyl‐3‐trimethylammonium‐propane (chloride salt) (DOTAP), 1,2‐dipalmitoyl‐sn‐glycero‐3‐phosphocholine (DPPC), Cholesterol, and 1,2‐distearoyl‐sn‐glycero‐3‐phosphoethanolamine‐N‐[amino(polyethyleneglycol)‐2000] (DSPE‐PEG2000) lipids (in molar ratio 40:40:10:10) were dissolved at 10 mg mL^−1^ in ethanol. For FRET LNPs, Egg Liss NBD and Rhodamine B PE lipids were added at a molar ratio of 0.5 each. Cy5‐labeled PC lipids at a 0.05% molar ratio were added to the lipid mixture for fluorescent labeling of LNPs. PBS buffer was used as an aqueous phase for the formulation. A SHM microfluidic device generated LNPs with a flow rate ratio of 1:3 (Lipid in ethanol: aqueous PBS).^[^ ^70^ ^]^ Later, the LNPs were dialyzed against PBS to remove ethanol. LNPs were then characterized for their size and zeta potential using DLS and NTA.

### Fabrication of Droplet Squeezer

The device was initially designed using AutoCAD (USA) software, and a 50 µm height SU‐8 master mold was fabricated through conventional photolithography. A droplet generator was created with two sample inlets and one oil inlet, followed by a short mixing unit and 40 rows of droplet squeezing structures, each row had 10 squeeze units with a gap of 4 µm. To construct the droplet squeezer, a Polydimethylsiloxane (PDMS; Corning, USA) mixture, prepared at a 10:1 base‐to‐curing agent ratio, was poured over the master mold and cured at 65 °C overnight. Once cured, the PDMS slab was removed, cut to the desired dimensions, and perforated with 1 mm holes using a biopsy punch (Miltex, USA) to create fluidic ports. The PDMS device was then plasma bonded to glass slides and cured at 65 °C overnight to ensure a leak‐proof seal.

### Droplet Generation and Optimization of Droplet Squeezing Device

We generated droplets to optimize the droplet generator device concerning the squeeze number. EVs and LNPs at 1 × 10^10^ particle mL^−1^ each were pumped in with both at a flow rate of 0.4 mL h^−1^ and oil at 2.5 mL h^−1^ forming droplets. Sequentially, we squeezed the droplets with EV and LNPs encapsulated through devices with 10, 20, 30, and 40 squeezing rows with a total flow rate of 3.3 mL h^−1^. Similarly, various devices with a squeezing gap of 2, 4, 8, and 10 µm were generated and ran droplets across these devices to assess fusion activity between EVs and LNPs. Using 40 squeezes and a squeeze channel length of 4 µm, we evaluated the effect of flow using the total flow rates ranging from 1–8 mL h^−1^.

### COMSOL Simulations

The droplet generator's AutoCAD design file was imported into COMSOL Multiphysics software and extruded 50 µm to mimic the device. Both inlet and outlet boundaries are defined with pressure at zero and initial velocity equal to the experimental flow rate velocity of 4.37 × 10^−11^ m^3^ s^−1^ (3.3 mL h^−1^). Study solutions were computed with a finer mesh setting. The most critical shear rate parameter was calculated using the data plots generated from the study solution of velocity and pressure plots.

### Fluorescent Imaging of EV Hybrids

To determine the fusion via fluorescence, EVs, and LNPs were stained with different fluorophores. EV surface proteins were labeled with NHS‐PEG4‐AF555, and LNPs were stained by incorporating Cy5‐DPPC lipid during formulation. Excess NHS‐PEG4‐AF555 was removed using a 40 kDa Zeba column (Thermo Fischer Scientific). EV hybrids generated from droplet squeezing were imaged in TRITC and Cy5 channels using an IX83 inverted fluorescence microscope with a 40x objective (Olympus, Japan).

### Sample preparation for Cryo‐ET

Samples were plunge frozen using a Leica EM GP2 (Leica Microsystems, Wetzlar, US) using a liquid ethane‐propane‐mixture as the cryogen. The cryogen was in turn cooled by liquid nitrogen and held at −180 °C. 4 µL of each sample was added to EM grids (Quantifoil R2/2 carbon membrane on Cu 200 mesh), blotted for 3–5 sec from the front and quickly plunged into the cryogen. Sample concentrations: LNP – 3 × 10^10^ particles mL^−1^; EV – 3 × 10^10^ particles mL^−1^; and hybrids: 5 × 10^10^ particles mL^−1^ at LNP:EV ratio of 1:1 particles.

### Cryo‐ET Image Acquisition

Cryo‐ET was performed on a ThermoFisher Krios G3i 300 keV field emission gun cryo‐TEM as mentioned previously.^[^ ^71^ ^]^ Images were collected using the SerialEM software on a K3 direct electron detector (Gatan Inc., Pleasanton, CA, USA) that operated in electron‐counted mode.^[^ ^72^ ^]^ After initially assessing the samples at lower magnifications for suitability of ice thickness and lipid membrane integrity, tilt series were collected with a span of 120^0^ (−60° to +60°; dose‐symmetric scheme) with 2° increments at a magnification of 33 000X (with a corresponding pixel size of 2.67 Å) and a defocus value of −5 µm. The cumulative dose of each tilt series was close to 100 e^−^/Å.^2^ The Gatan Imaging Filter (Gatan Inc., Pleasanton, CA, USA) operated with a slit width of 20 eV helped to increase contrast by removing inelastically scattered electrons (Krivanek et al.). Once acquired, tilt series were aligned and reconstructed into tomograms using the IMOD software package (Kremer et al.).^[^ ^73^ ^]^ Slices through tomograms were used to analyze LNP fusion with EVs and to generate the representative images included in this manuscript.

### FRET Studies for Fusion Activity

Fusion activity was studied using FRET. Briefly, a FRET pair of 0.5 mol% each of NBD‐PE (donor) and Rhodamine B‐PE (acceptor) lipids were incorporated in the lipid mixture. LNPs formulated with these lipids were employed to perform FRET studies. FRET pair‐incorporated LNPs were fused with NK or A431 cell‐derived EVs using the DASH technique. The fluorescence signal from the fused vesicles was measured using a spectrophotometer (TECAN Spark Control, Switzerland). The fusion activity was assessed from the ratio of NBD/ RhB fluorescence. For this measurement, the samples were excited at 460 nm and emissions were recorded at 530 and 585 nm. The increase in NBD/RhB ratio directly correlates with an increased fusion of LNPs and EVs.

### Conventional Techniques Used for the Synthesis of EV Hybrids

Various conventional EV hybrid generation techniques were employed for comparing fusion against droplet squeezing. Bulk mixing included mixing 1 × 10^10^ particles mL^−1^ equal ratios of EVs and LNPs and mixing the sample at 37 °C for 1 h at 700 rpm. Similarly, the avanti mini extruder was employed to fuse EVs and LNPs with a 0.2 µm Nuclepore Track‐Etch membrane (Cytiva). A minimum of 15 rounds of extrusion was applied to generate EV hybrids from a 1:1 ratio of EV:LNP at 1 × 10^10^ particles mL^−1^ each. For the freeze‐thaw method, we mixed EVs and LNPs in equal ratios (1 × 10^10^ particles mL^−1^) in a falcon tube and plunged into liquid nitrogen for 30 s, followed by a 15‐min thaw at room temperature.^[^ ^74^ ^]^ At least five cycles of freeze‐thaw were performed.

### Cargo Loading of EV Hybrids

For nucleic acid loading, the EGFP plasmid or random siRNA were mixed with 50 mm citrate buffer and lipid ethanol solution in a microfluidic SHM device. To remove ethanol, LNPs loaded with nucleic acids were dialyzed overnight with a membrane molecular weight cutoff of 300 kD. For small molecule loading, chemo drugs were mixed in ethanol while preparing a lipid mixture. Later, the drug/lipid solution and PBS were mixed in a microfluidic device to generate drug‐loaded LNPs. LNPs were dialyzed against the 300 kD molecular weight cutoff membrane to remove ethanol and unloaded drug.

### Nano‐Flow Cytometry

AF555‐stained EVs and Cy5‐labeled LNPs were used to generate EV hybrids through droplet squeezing. EV hybrids, along with single stain and unstained controls, were analyzed using nano‐flow cytometry (NanoFCM, Inc., Xiamen, China) from the University of Pennsylvania School of Veterinary Medicine Extracellular Vesicle Core. A similar BD cyto‐FLEX nano flow cytometer was employed to assess EV hybrids with dual staining from EVs and LNPs. All samples were diluted to achieve a particle count with the range between 2000–12 000/min to remove any swarming effects. A total of 50 000 events were collected, and dot plots were generated using NanoFCM software (NanoFCM Profession V1.0) and FlowJo software (USA).

### Cell Immunostaining

To determine the DR5 expression in A375 melanoma cell lines, immunostaining with anti‐DR5 Ab and secondary fluorescent Ab was employed. Briefly, DR5 KO, WT, and DR5 OExp A375 melanoma cells were stained with anti‐DR5 monoclonal antibody after blocking with bovine serum albumin. Later, cells were washed and goat anti‐human IgG secondary Ab (Invitrogen) tagged with a fluorophore was used for staining the cells. Then, cells were washed 3X PBS and ran in BD LSR II (Penn cytomics and cell sorting laboratory, University of Pennsylvania). Flow cytometry data was analyzed using Flowjo software (Flowjo LLC, USA).

### Immunoelectron Microscopy of EV Hybrids

For immunoelectron microscopy, EVs or EV hybrids suspended in PBS were deposited onto formvar carbon‐coated nickel grids, then blocked and incubated with F(ab′)2 Fragment Goat Anti‐Human IgG. This was followed by incubation with gold‐conjugated goat anti‐biotin (10 nm, electron microscopy grade) from Electron Microscopy Sciences (PA, USA). After each staining step, the samples underwent 5X PBS washes before being contrast‐stained with 2% uranyl acetate. A similar staining protocol was performed for LNPs, excluding the F(ab′)2 antibody staining. The EVs, LNPs, and EV hybrids were then imaged using a JEM‐1011 transmission electron microscope.

### EV Hybrid Uptake Studies

EVs, LNPs, and EV hybrids were given to A375 melanoma cell lines with varying DR5 expression levels, including KO, WT, and OExp. EVs were labeled with AF555, while LNPs were labeled with Cy5. For this experiment, cells were seeded in an 8‐chambered cover glass system (Cellvis, USA). The cells were treated with 1 × 10^10^ particles mL^−1^ of either EVs, LNPs, or EV hybrids. After a 4‐h incubation, cells were washed 3X PBS, stained with DAPI (Invitrogen, USA) for nuclear localization and CellTracker Green (Invitrogen, USA) for cytoplasm visualization. Fluorescence images were captured using an IX83 inverted fluorescence microscope (Olympus, Japan), focusing on the mid‐section of cells with nuclei in focus to analyze internalized EV hybrids.

Cell uptake images were processed using a custom‐designed CellProfiler pipeline. Briefly, image intensities were rescaled, and cell boundaries were identified based on CellTracker Green staining. EVs and LNPs were segmented based on pixel size and granularity. Colocalization of EVs and LNPs was determined using the *RelateObjects* module, which pairs identified particles based on spatial proximity. Once colocalized EVs and LNPs were identified, these particles were overlaid onto the cell mask derived from boundary identification via Cell Tracker Green stain. The number of internalized particles per cell was quantified, and the average particle count per cell across three images was calculated and plotted.

### LC‐MS Protocol for LNP Drug Concentration Measurement

Ulixertinib concentrations were determined by LCMS analysis of ethanol‐lysed samples. Ulixertinib calibration curves were generated by running samples containing 10 ng mL^−1^ to 100 µg mL^−1^ and then integrating across the 254 nm absorbance ulixertinib peak. The calibration curve was measured every 6 months to account for drift. Samples were analyzed using a 1260 Agilent Infinity II HPLC system equipped with an SB C18 2.1 × 50 mm, 1.8 mm column with a mobile phase consisting of H_2_O (solvent A), acetonitrile (solvent B) and a gradient composed of 5% B at 0 min to 95% B at 10 min, then to 5% B at 12 min with a flow rate of 0.4 mL min^−1^. Experimental samples were run under identical conditions; the 254 nm UV absorbance was integrated across the ulixertinib peak and compared to calibration curves to determine the experimental concentration. Known concentrations of LNPs were lysed with 100% ethanol and measured by LC‐MS to find drug concentrations of ulixertinib loaded inside the LNPs. A similar protocol was employed to measure ravoxertinib concentrations. Loading efficiencies were calculated using the following equation.

(1) $$Loading\;efficiency=\frac{Drug\;in\;lysed\;mg\;of\;LNPs}{Total\;drug\;used\;for\;formulation\;}\;X\;100$$

### Lyophilization Studies of EV Hybrids

EV hybrids generated from the DASH technique were stored at 4 °C. EV hybrids from the stored stock solution were divided into two groups, one without any cryoprotectant and the other mixed with 8.5% w/v sucrose.^[^ ^33^ ^]^ Both groups were lyophilized using a Labconco FreeZone 2.5 freeze‐drying system. Samples were collected after overnight lyophilization. Later, similar initial volumes of PBS were used to resuspend lyophilized sample powders. The resuspended lyophilized samples’ size and zeta potential were measured using DLS.

### Drug Dosage Studies

Two ERK inhibitor drugs, Ulixertinib (BVD‐523) and Ravoxertinib (GDC‐0944), which have shown potency against melanoma, were employed to load into LNPs. Both hydrophobic drugs were mixed with lipids in ethanol and were injected into a microfluidic SHM device along with PBS to generate drug‐loaded LNPs. LNPs were dialyzed against a 10 kD molecular weight cutoff dialysis membrane overnight. Luciferase^+^ A375 cells at 20 000 cells well^−1^ were seeded without FBS until attached. Later LNPs and free drug of ulixertinib and ravoxertinib were given to A375 melanoma cells with a DMEM concentration of 100 nm in 1% FBS and 1% pen‐strep. After 3 days, cells were washed with PBS and lysed with a lysis reagent of 45 µL. Luciferase assay reagent (100 µL per well) was added to each well and read using a TECAN SPARK spectrophotometer (Luciferase Assay system, Promega).

### Antibody Conjugated LNP Preparation

As previously described, anti‐DR5 monoclonal Abs were conjugated to the surface of LNPs using maleimide‐thiol click chemistry.^[^ ^75^, ^76^ ^]^ Briefly, ulixertinib‐loaded LNPs were synthesized, incorporating DSPE‐PEG5k‐Mal (0.5 mol%) into the lipids. Anti‐DR5 monoclonal Abs were functionalized by reacting with SATA (N‐succinimidyl S‐acetylthioacetate) in DMSO (15 mg mL^−1^). This reaction targeted primary amines in the antibody. This reaction was carried out in PBS containing 1 mm EDTA at a protein concentration of 10 mg mL^−1^. The reaction was incubated at room temperature for 1 h, after which unreacted SATA was removed using Zeba desalting columns (7 kDa molecular weight cutoff, Thermo Fisher).

To deprotect the sulfhydryl (‐SH) groups, a 10% (v/v) hydroxylamine solution was added, containing 50 mm sodium phosphate, 25 mm EDTA, and 0.5 m hydroxylamine HCl. The mixture was incubated at room temperature for 2 h, followed by another desalting step to remove excess reagents. The thiolated Abs were mixed with maleimide‐functionalized LNPs at a 2.5‐fold molar excess (Ab: maleimide) and incubated at room temperature for 2 h. Conjugation was completed by overnight dialysis against PBS using a 300 kDa molecular weight cutoff dialysis membrane to remove unbound Abs and reaction byproducts.

### In Vitro Cancer Killing Assay

LNPs loaded with ulixertinib were fused with DR5 scFv expressing EVs derived from CAR‐NK92 cells to generate ulixertinib‐loaded EV‐LNP hybrids. EVs were loaded with ulixertinib via sonication at 37 °C for 30 min. Luciferase^+^ WT A375 melanoma cells were seeded at a density of 40 000 cells per well in a 96‐well plate and maintained in serum‐free conditions until attachment. Both 2D cell cultures (in 96‐well plates) and 3D spheroid models were treated with ulixertinib‐loaded EV hybrids with control groups including EVs, ulixertinib‐loaded EVs, ulixertinib‐loaded LNPs, and DR5 Ab‐conjugated ulixertinib‐loaded LNPs. An equivalent drug concentration of 500 nm was maintained across all groups. The drug concentration in LNPs, EVs, and EV hybrids was quantified by lysing the particles with 2% Triton X‐100, followed by LCMS analysis. After 72 h, 2D and 3D cultures were lysed using a lysis reagent and mixed with a luciferase reagent to measure melanoma cell luciferase activity using a TECAN SPARK plate reader (Luciferase Assay System, Promega). For 3D spheroid assays, spheroids were carefully transferred to 96‐well plates via pipetting to minimize contamination from the agarose gel layer. For the LIVE/DEAD imaging of spheroids, after 3 days, GFP^+^ melanoma cells within the spheroid structures were subjected to imaging across various experimental groups to assess live cell populations. To differentiate dead cells, propidium iodide was incorporated into the culture medium at a concentration of 10 µg mL^−1^, facilitating the visualization of necrotic and apoptotic cells.

For determining the baseline toxicity of free ulixertinib and anti‐DR5 Ab, we performed a cytotoxicity study in A375 melanoma cell lines. Anti‐DR5 Ab, free ulixertinib, free ulixertinib + anti‐DR5 Ab, ulixertinib‐loaded EV hybrids with an equivalent drug concentration of 100 nm ulixertinib were given to A375 cells (40 000 cells well^−1^ seeded in 1% FBS). The DR5 Ab dose was calculated based on the average number of DR5 scFV from TEM images of EVs. After 3 days, cells were washed, lysed, and treated with luciferase reagent. Later, luminescence was captured using a plate reader (TECAN).

RNA was extracted using TRIzol reagent (Invitrogen) to evaluate gene expression following treatment in 2D cell experiments. Complementary DNA (cDNA) was synthesized using the iScript cDNA synthesis kit (Bio‐Rad). Apoptosis‐related genes, including Caspase‐8, Caspase‐3, STAT‐3, Bax, BIRC5, and Cyclin D1, were analyzed to assess the effects of EV hybrid treatment (primer sequences listed in Table S1, Supporting Information). A housekeeping gene of RPL13A was employed to calculate the fold change expression of various genes. cDNA and gene‐specific primers were combined with PowerTrack SYBR Green Master Mix (Applied Biosystems; A46109) and nuclease‐free water (Integrated DNA Technologies) for gene expression studies. Quantitative PCR (qPCR) was performed in 10 µL reactions, running for 40 cycles on a QuantStudio 3 Real‐Time PCR system (Applied Biosystems).

## Conflict of Interest

The authors declare no conflict of interest.

## Author Contributions

U.C. and J.K. conceived and designed the project. U.C., S.K.M., S.L., A.F.D.C., J.L., Y.R., X.Z., R.T.M. helped with the experiments. U.C., Y.W.C., M.S., X.X., and J.K. discussed the data and helped with the manuscript outline. The manuscript was written through the contributions of all authors. All authors have given approval to the final version of the manuscript.

## Supporting information



Supporting Information

Supplemental Video 1

## Data Availability

The data that support the findings of this study are available from the corresponding author upon reasonable request.
