# Supplementary material for: Droplet Squeeze Microfluidic Platform for Generating Extracellular Vesicle Hybrids for Drug Delivery
Source: Small. 2025 Aug 7;21(37):2503807. doi: 10.1002/smll.202503807 (PMC12444820; doi:10.1002/smll.202503807)

***Supporting Information***

**Droplet Squeeze Microfluidic Platform for Generating Extracellular Vesicle Hybrids for Drug Delivery**

Uday Chintapula^a^, Shujing Liu^a^, Andres Fernandez Del Castillo^b^, Jianhua Lim^c^, Yoonho Roh^a,d^, Shrawan Kumar Mageswaran^b^, Xiaogang Zhang^a^, Renee-Tyler T. Morales^c^, Mark A. Sellmyer^b,e^, Yi-Wei Chang^b^, Xiaowei Xu^a^, Jina Ko^a,c*^

**^a^** Department of Pathology and Laboratory Medicine, University of Pennsylvania, Philadelphia, PA, 19104, USA

**^b^** The Department of Biochemistry and Biophysics, Perelman School of Medicine, University of Pennsylvania, Philadelphia, PA, USA

**^c^** Department of Bioengineering, University of Pennsylvania, Philadelphia, PA, 19104, USA

**^d^** Department of Energy and Chemical Engineering, Incheon National University, 119 Academy-ro, Yeonsu-gu, Incheon 22012, Republic of Korea

**^e^** Department of Radiology, Perelman School of Medicine, University of Pennsylvania, Philadelphia, PA, USA

*Jina Ko, PhD

Department of Bioengineering

Department of Pathology and Laboratory Medicine 440 Curie Blvd, Philadelphia, PA, 19104

713-628-0283

[Jina.Ko@pennmedicine.upenn.edu](mailto:Jina.Ko@pennmedicine.upenn.edu)

**Figure S1. Characterization of LNP lipid composition**. **a)** Lipid composition utilized for generating LNPs that fuse with EVs: NBD and RhB fluorescent probes tagged with DSPE were added for FRET studies. DLS measurements were conducted with varying concentrations ranging from 0-10% DSPE-PEG-2K to assess their **b)** size and **c)** zeta potential.


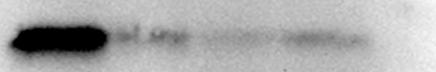

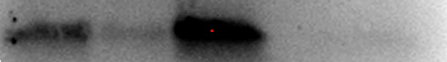


CD9

CD81

NKEVs

NK WCL

T-cell WCL

25 kDa

20 kDa

Calnexin

NKEVs

NK WCL

90 kDa

a

b


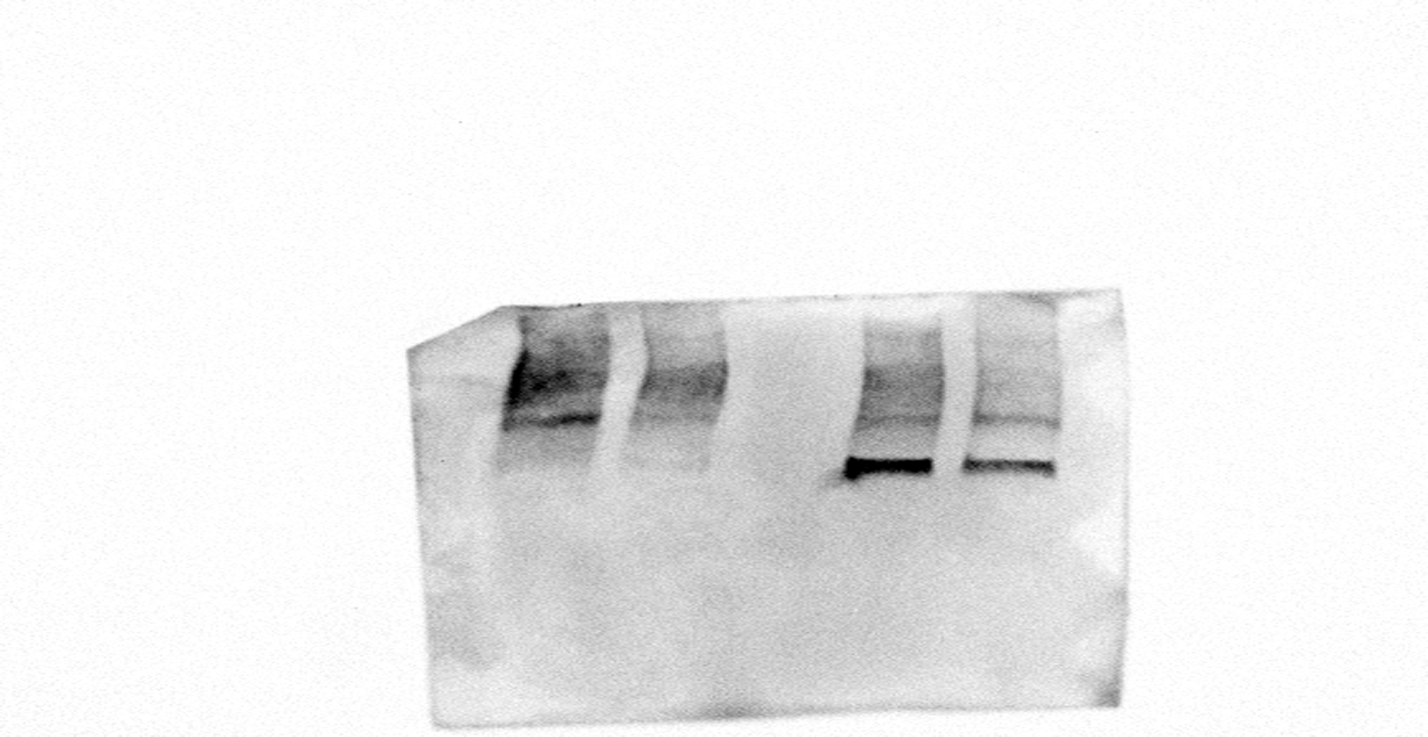

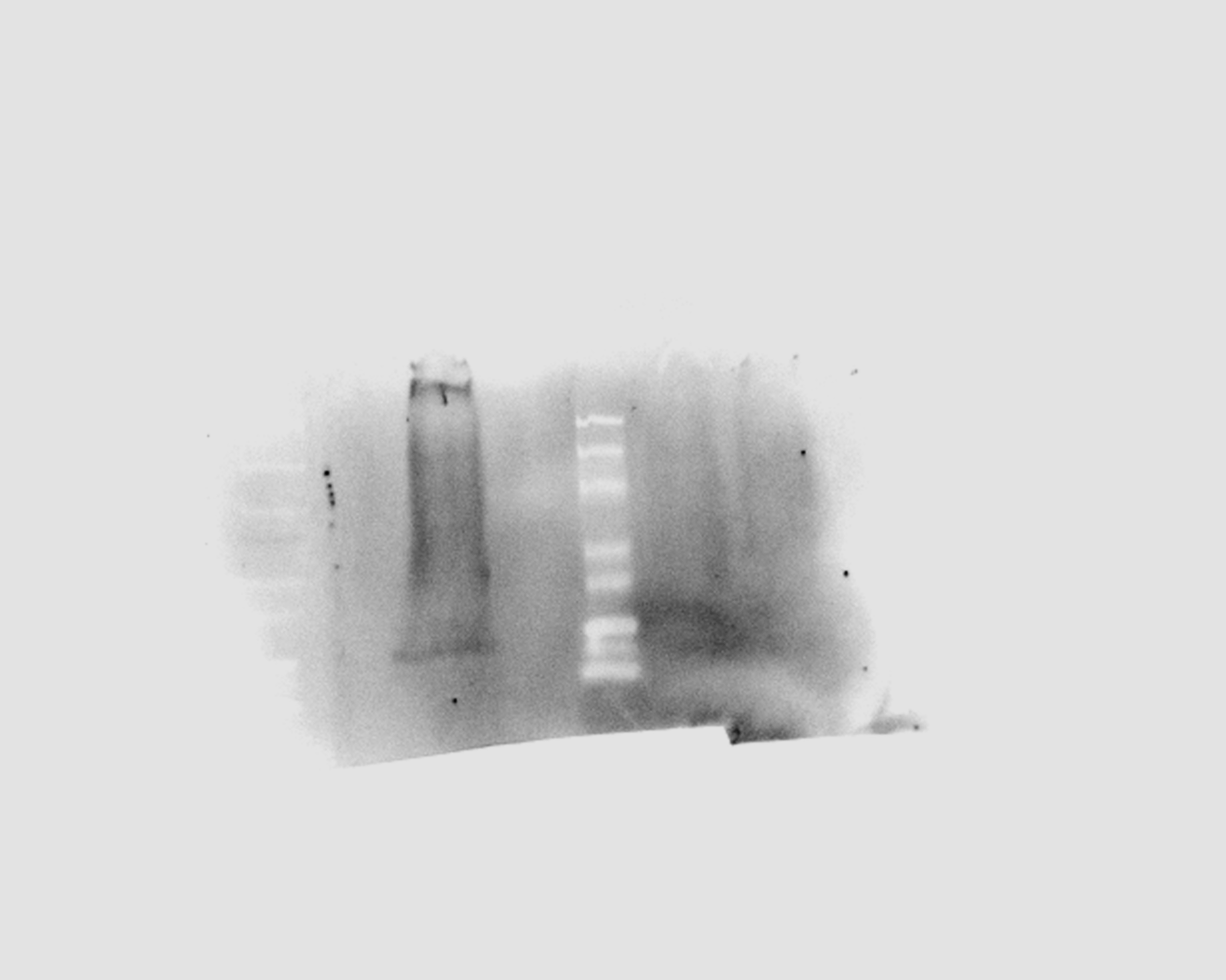


TSG101

NKEVs

NK WCL

50 kDa

c

**Figure S2. Western Blot of CAR-NK92 sEVs**. **a)** Western blot of CD9 and CD81 expression in sEVs derived from CAR-NK92 cells, along with activated T cell control and NK cell whole cell lysates (WCL). EVs were lysed using RIPA buffer supplemented with protease inhibitor cocktail, and 10–20 µg of total protein was loaded per lane. CD9 detection was performed under non-reducing conditions, while CD81 detection was carried out under reducing conditions. **b)** Calnexin expression in CAR-NK92-derived sEVs and corresponding whole cell lysate was assessed under reducing conditions. **c)** TSG101 expression in CAR-NK92-derived sEVs and corresponding whole cell lysate was assessed under reducing conditions.

**Figure S3. a**) A microfluidic device is configured to generate EV hybrids through droplet squeezing. **b**) Illustration depicting droplet squeezing through the specialized microfluidic geometry designed for the DASH method.

a

b

**Figure S4. Lyophilization of EV hybrids. a.** DLS measured size and PDI of EV hybrids stored at 4^0^C, resuspended after lyophilization with and without 8.5% w/v sucrose. **b.** DLS measured zeta potential of resuspended lyophilized EV hybrids with and without 8.5% sucrose along with 4^0^C stored hybrid control. **FD**: Freeze Drying

**Figure S5**. **a)** Small particle flow cytometry dot plot data with EV hybrids (P1 quadrant) generated from NHS-PEG-AF488-stained EVs and Cy5-labeled LNPs at EV: LNP ratios of 10:1, 1:1, and 1:10. **b)** %EV hybrids calculated for various ratios using the equation below.

$$\% EV hybrids=\frac{co localized population in P1 quadrant}{EVs \left( P2 \right)+LNPs (P4)}$$

**Figure S6.**  EV hybrid generation utilizing adherent (A431 cells) and suspension (NK92 cell EVs) was achieved. Various techniques were employed, including bulk mixing, droplet mixing, and droplet squeezing. The droplet squeezing method showed higher fusion activity, while suspension cells EVs have comparatively higher fusion activity.

**Figure S7. a**) Illustration demonstrating DR5 scFv staining of EV hybrids formed from the fusion of Cy5-labeled LNP and NHS-PEG-AF555 labeled DR5 sEVs, using FITC F_ab_ Ab. **b**) The contour plot from the small particle flow cytometer indicates a double positive population of EV hybrids (purple). **c)** Isolated double-positive populations were gated based on their FITC fluorescence, along with control groups of LNPs, EVs, and EV hybrids that did not undergo any FITC F_ab_ Ab staining.

**Figure S8.** Fluorescent images demonstrate the uptake of DR5 scFv EVs in yellow (first column) and Cy5 labeled LNP in red (second column) across various DR5 receptor-expressing melanoma cells A375 (knockout, wild type, and overexpressing cells).

**Figure S9.** The uptake of EV hybrids in cells was quantified using CellProfiler software. A detailed pipeline for quantifying EV hybrid uptake in each cell was established.

**Figure S10.** DLS measurements indicate the size, zeta potential, and PDI of ravoxertinib-loaded LNPs and EV hybrids created from the fusion of these LNPs with DR5 EVs.

**Figure S11. a)** Schematic illustration of maleimide-thiol click chemistry demonstrating the process of antibody conjugation. Thiolated antibodies were efficiently conjugated to maleimide-functionalized LNPs. **b)** To confirm the successful conjugation of human DR5 antibodies on LNPs, the nanoparticles were stained with secondary antibodies: mouse IgG and human IgG. Staining results indicate the presence of functional DR5 antibodies on the LNPs. **c)** A range of molar ratios of antibodies to maleimide (in LNP) was evaluated to determine the optimal conditions for antibody conjugation. Fluorescently labeled secondary human IgG was utilized for staining to validate the conjugation of DR5 antibodies.

**Figure S12. a**) Cell viability was evaluated in luciferase-positive (Luc+) A375 melanoma cells subjected to treatment with ulixertinib, administered as a free drug or encapsulated within EVs, LNPs, DR5 antibody-conjugated LNPs, and hybrid EV formulations at a concentration of 500 nM. **b**) A heat map illustrates the gene expression profiles of six apoptosis-related genes, contrasting the effects of ulixertinib treatment as a free drug, LNPs, and hybrid EV formulations. Gene expression levels were normalized to the reference gene RPL13A. **c**) The evaluation also included cell viability from spheroids comprising Luc+ A375 melanoma cells and luciferase-negative BJ human fibroblasts, treated with 2.5 µM ulixertinib, either as a free drug or delivered through EVs, LNPs, DR5 Ab-conjugated LNPs, and hybrid EVs.

**Figure S13. Baseline toxicity of free ulixertinib and DR5 Ab treatments**. A375 melanoma cells were treated with DR5 Ab molecules at a molar concentration equivalent to EV hybrids loaded with 100 nM of ulixertinib (ulix). Treatment groups included DR5 Ab alone, free ulix (100 nM), a combination of ulix at 100 nM with DR5 Ab, and EV hybrids loaded with ulix (100 nM). Cells were incubated for 72 hours, and cell viability was assessed using a luciferase-based assay.

**Table S1.** Primers for qPCR

| **Genes** | **Forward primer (5’-3’)** | **Reverse primer (5’-3’)** |
| --- | --- | --- |
| RPL13A | CTCAAGGTGTTTGACGGCATCC | TACTTCCAGCCAACCTCGTGAG |
| Caspase-8 | AGAAGAGGGTCATCCTGGGAGA | TCAGGACTTCCTTCAAGGCTGC |
| Caspase-3 | GGAAGCGAATCAATGGACTCTGG | GCATCGACATCTGTACCAGACC |
| STAT-3 | CTTTGAGACCGAGGTGTATCACC | GGTCAGCATGTTGTACCACAGG |
| BIRC5 | CCACTGAGAACGAGCCAGACTT | GTATTACAGGCGTAAGCCACCG |
| Bax | TCAGGATGCGTCCACCAAGAAG | TGTGTCCACGGCGGCAATCATC |
| Cyclin D1 | GATGCCAACCTCCTCAACGA | ACTTCTGTTCCTCGCAGACC |

**Video V1.** Movie showing droplets squeezing through the squeeze gaps on DASH device


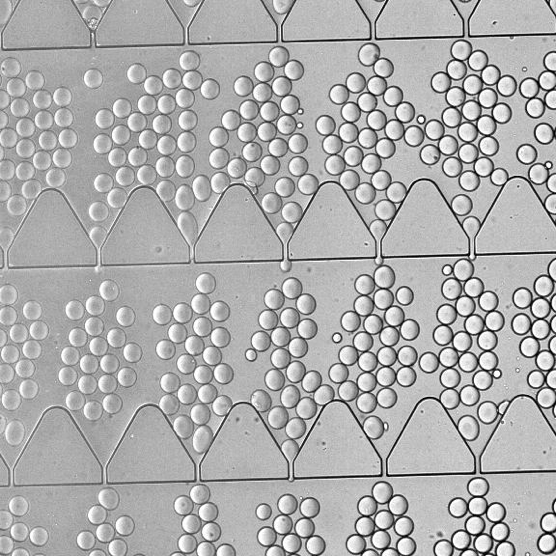

Supplement: Supplementary file 1 — Supporting Information [file SMLL-21-2503807-s001.docx]
